# Overcoming Oral Mucosal Barriers: Next-Generation Oromucosal Formulation Strategies for Efficient Therapy of Oral Diseases

**DOI:** 10.3390/jfb17100491

**Published:** 2026-09-29

**Authors:** Xincheng Lin, Yichun Zheng, Ziqiao Zhong, Lu Gan, Chuanbin Wu, Xin Pan, Wenhao Wang, Ying Huang

**Affiliations:** 1 State Key Laboratory of Bioactive Molecules and Druggability Assessment, Guangdong Basic Research Center of Excellence for Natural Bioactive Molecules and Discovery of Innovative Drugs, College of Pharmacy, Jinan University, Guangzhou 511443, China; 2 School of Pharmaceutical Science, Sun Yat-sen University, Guangzhou 510006, China; panxin2@mail.sysu.edu.cn

**Keywords:** drug delivery system, biomaterials, oral mucosal diseases, local administration, delivery barriers, oromucosal adhesion, oromucosal penetration, oral biomechanics

## Abstract

Oral mucosal diseases often lead to structural damage and functional impairment due to high recurrence and malignant progression. Current therapeutic outcomes remain unsatisfactory owing to the complex oral microenvironment. Recent reviews have covered individual aspects of oral mucosal drug delivery, such as bioactive materials, mucoadhesive systems, and nanocarriers, but few have considered all four interrelated dimensions, including saliva-mediated adhesive barriers, epithelial permeability, biomechanical adaptability, and translational bottlenecks, in an integrated manner. The present review offers an attempt to consolidate these dimensions into one framework, aiming to provide readers with a more comprehensive perspective on the challenges inherent in this field. To underpin this framework, we conducted a structured literature search covering Web of Science (2015–2026), with predefined inclusion/exclusion criteria, followed by thematic synthesis. We first summarize the clinicopathological features and current treatment landscape of major oral mucosal diseases. We then dissect three critical barriers to transmucosal delivery: adhesive interference by salivary flow, epithelial tight junction resistance, and dynamic biomechanical forces (e.g., chewing, tongue movement). Drawing on saliva and mucosa interfacial physics, we rationalize the need for wet-adhesive material design and survey diverse mucoadhesive polymers and formulations, detailing their binding mechanisms. For epithelial penetration, we highlight design principles of microneedles and nanocarriers while critically discussing their dual-edged safety–efficacy profiles. To cope with the highly deformable oral biomechanical environment, we propose mechanical property matching and asymmetric structural designs. Finally, we outline key translational bottlenecks and future breakthroughs. This review offers an integrated, barrier-informed guide for next-generation oromucosal formulations.

## 1. Introduction

The oral mucosa functions as a barrier for humans, integrating protection, absorption, secretion, and sensation through a dynamic stratified architecture composed of the epithelium, lamina propria, and submucosa. In addition to constituting a physical, chemical, and immunological barrier, this tissue maintains local homeostasis through continuous interactions with saliva and the oral microbiota. However, this delicate equilibrium will be disrupted by many pathological conditions, which subsequently give rise to oral diseases. Such conditions impose a substantial global health burden. Recurrent aphthous ulcers (RAU) are one of the most common causes of oral ulcers, thereby compromising eating and speaking [1]. Oral squamous cell carcinoma (OSCC) ranks as the sixth most common cancer worldwide, accounting for more than 90% of all oral malignancies [2,3,4,5]. Oral mucositis exhibits an extremely high occurrence among patients undergoing radiotherapy or chemotherapy [6,7], often leading to treatment interruptions and increased healthcare costs [8]. Furthermore, chronic inflammation and immune dysregulation in diseases such as oral lichen planus (OLP) and oral leukoplakia (OLK) create a microenvironment that may promote malignant transformation [9,10], underscoring the urgent need for effective and sustained local interventions.

Current pharmacotherapeutic strategies for managing oral mucosal diseases rely primarily on systemic and topical routes of administration. Systemic delivery, typically via oral tablets or parenteral injection, can attain therapeutic concentrations at the target site. However, its efficacy is frequently compromised by hepatic first-pass metabolism, limited and variable local drug concentration, and a substantial risk of systemic adverse effects [11]. Topical formulations such as gels, ointments, sprays, mouthwashes, and patches provide the theoretical advantage of direct drug application with minimal systemic exposure [12]. Several of these products have received clinical approval and have attracted increasing attention in oral medicine. However, their clinical efficacy is severely limited by the unique physiological barriers of the oral environment. Saliva retained in the oral cavity not only hinders mucosal drug delivery, but the inherently present oral mucosal tissue also forms both an adhesive barrier and a permeation barrier [13,14]. The continuous secretion of saliva creates a dynamic fluid layer that rapidly dilutes and clears conventional formulations, while the salivary film thickness and the presence of mucins hinder adhesive interactions between the formulation and the target tissue [14]. The stratified oral epithelium poses two obstacles for drug absorption. Firstly, its lubricating glycocalyx and membrane-bound mucins create an adhesion barrier, making it difficult for drugs to attach. Secondly, the tight junctions and intercellular lipids form a permeation barrier, which actively restricts drug diffusion into deeper layers [15]. Furthermore, incessant mechanical activities such as chewing, speaking, and moving the tongue subject formulations to shear forces and easy detachment, further shortening their stay on the oral mucosa [15]. As a result, the low local drug concentration of most conventional topical formulations requires frequent dosing, leading to unstable clinical outcomes [16].

In recent years, the limitations of conventional oral mucosal therapies have motivated increasing exploration of advanced drug delivery systems, including mucoadhesive films, hydrogels, microneedle patches, among others. To gain a comprehensive understanding of the development status in this field and thereby propose more forward-looking formulation strategies, this review systematically outlines oral diseases and delivery barriers, proposing key points for next-generation oromucosal formulation design. We clarify the anatomical and pathophysiological foundations of oral mucosal diseases while analyzing the current problems faced by existing commercial formulations. Additionally, we systematically examine the major barriers that prevent drug delivery to the oral mucosa. Then, we conclude with three specific strategies to overcome these obstacles: adhesive strategies, penetrative strategies, and formulation optimization strategies. Finally, we discuss significant challenges in applying next-generation oromucosal formulations to oral diseases and future research directions. Through comprehensive and integrated analysis, this review aims to provide new perspectives for oromucosal formulation design, offering theoretical support for developing personalized oral care platforms and achieving precision treatment in oral medicine.

## 2. Oral Mucosal Diseases

Clinically, oral mucosal diseases can be categorized into three major groups, namely oral tumors and related disorders, inflammatory and immune-mediated diseases, and infectious diseases. Among these, oral tumors and related disorders commonly include oral leukoplakia and oral squamous cell carcinoma. Inflammatory and immune-mediated oral mucosal diseases mainly comprise oral ulcers, treatment-related oral mucositis (such as radiation-induced oral mucositis (RIOM) and chemotherapy-induced oral mucositis (CIOM)), and oral lichen planus. Infectious oral mucosal diseases are divided into fungal oral candidiasis (OC) and viral infections such as herpes simplex. To facilitate a clearer understanding and comparison of the four readily confused oral mucosal diseases, including recurrent aphthous ulcers, diabetic oral ulcers, treatment-related oral mucositis, and oral leukoplakia, we have summarized and delineated their disease characteristics, pathogenetic mechanisms, primary oral mucosal barriers, and clinical therapeutic needs in Table 1. Oral leukoplakia, a common oral potentially malignant disorder, exhibits a well-defined potential for malignant transformation. It typically arises from the interaction between environmental carcinogens and genetic susceptibility, which leads to dysregulated cell proliferation and chronic inflammation upon onset. Without intervention, leukoplakia may irreversibly progress into invasive carcinoma and typically does not resolve spontaneously. As the malignant endpoint of this disease spectrum, OSCC accounts for over 90% of oral cancer cases [17] and is recognized as a highly heterogeneous malignancy. Smoking and alcohol consumption are major risk factors for OSCC in most patients. Furthermore, sexually transmitted human papillomavirus, dysbiosis, Candida infection, nutritional deficiencies, and betel nut chewing are also associated with OSCC [18,19], demonstrating a pathogenesis strongly dependent on environmental and behavioral factors. Owing to its highly aggressive tumor behavior and significant treatment resistance, OSCC is prone to cervical lymph node metastasis and local recurrence, posing a major clinical challenge [20]. Advanced OSCC may metastasize to distant organs, primarily the lungs, bones, and liver, leading to rapid death within 3 to 6 months after diagnosis, with an extremely low 5-year overall survival rate of approximately 30% [21]. Inflammatory and immune-mediated oral mucosal diseases typically present with local pain, redness, swelling, and ulceration, severely impacting quality of life. RAU, commonly known as a canker sore, is a prevalent oral mucosal disease caused by a combination of multifactorial influences, including genetic predisposition, immune system dysregulation, nutritional deficiencies, hormonal changes, local oral trauma, microbial dysbiosis, psychological stress, and food sensitivities [22]. Currently, over 25% of the global population has experienced or is experiencing mucosal injuries [23]. Although non-contagious, RAU significantly affects oral health-related quality of life due to the painful and recurrent nature of the ulcers. When RAU coexists with systemic diseases, its pathological mechanism becomes more complex. Diabetic patients in a long-term hyperglycemic environment exhibit impaired immune cell function, leading to decreased defense against oral microorganisms [24]. Concurrently, microangiopathy limits the delivery of oxygen and nutrients, resulting in diminished tissue repair capacity and prolonged healing time [25]. In addition to oral ulcers, treatment-related oral mucositis represents another major oral mucosal disease. It is categorized into radiation-induced oral mucositis and chemotherapy-induced oral mucositis. These represent a widespread issue in cancer therapy, characterized by high incidence and delayed healing [26]. It is estimated that the prevalence of treatment-related oral mucositis is 40% in patients undergoing chemotherapy, 75% in those receiving high-dose chemotherapy, and exceeds 90% in patients receiving radiotherapy for head and neck cancers [27]. This self-perpetuating deterioration not only diminishes the patient’s quality of life but directly interrupts antitumor therapy in 20–30% of cases [28]. Oral lichen planus, as a model of T-cell-mediated autoimmune disease [29], is accompanied by a cytokine storm (IL-6, TNF-α). The disease manifests in several oral clinical forms, of which the three most frequent are the reticular (quiescent), erosive (active), and atrophic (late or post-lichenoid) types [30]. OC is the predominant type of infectious oral mucosal disease. As a common opportunistic fungal infection of the oral mucosa, it poses a significant health challenge for immunocompromised individuals, the elderly, and those with local predisposing factors. The pathogenesis of this disease is essentially the result of a disrupted balance between host defense and fungal pathogenicity, exhibiting strong biofilm-forming capacity (especially on artificial surfaces such as dentures and prostheses) and a trend toward multidrug resistance [31].

Turning to topical administration strategies (Table 2), their core advantage lies in the direct action of drugs on oral mucosal lesions, theoretically enabling targeted delivery and maximally avoiding systemic side effects. Commonly used topical formulations in clinical practice include gels, ointments, mouthwashes, sprays, and patches. As shown in Table 3, FDA approvals for local oral mucosal formulations from 2000 to 2026 have been limited in number. The approved products are predominantly conventional delivery systems, including mucoadhesive patches, oral rinses, gels, and buccal tablets, with approved indications mainly covering recurrent aphthous ulcers, oral mucositis, oropharyngeal candidiasis, and herpes labialis. Notably, despite the clinical burden of oral lichen planus and oral leukoplakia, no FDA-approved local formulations are available for these conditions, pointing to persistent unmet therapeutic needs. This regulatory landscape, together with the small number of approvals over more than two decades, reflects a translational gap and underscores the need for advanced delivery systems supported by robust clinical data. These can be loaded with drugs such as corticosteroids (e.g., triamcinolone, clobetasol) to potently suppress T-cell-mediated inflammatory cytokine storms (IL-6, TNF-α) for the treatment of inflammatory and immune-mediated oral mucosal diseases, serving as the cornerstone of treatment for RAU and OLP [40]. For treatment-related mucositis, although the use of mouthwashes containing analgesics (e.g., morphine), growth factors, or antimicrobials (e.g., chlorhexidine) is standard supportive care, these liquid formulations struggle to form an effective protective layer over ulcerated surfaces, especially when patient compliance is poor due to severe pain and dysphagia, limiting their evidence grade for prevention and treatment. Oral leukoplakia, as the most common oral potentially malignant disorder, is standardly treated with surgical excision. Drug therapy, such as topical application of retinoids or corticosteroids, is only attempted palliatively for inoperable cases. Its efficacy is uncertain, and it fails to effectively reverse the molecular pathways driving malignant transformation, thus contributing little to reducing the malignant transformation rate [41]. Topical administration of immunomodulators such as tacrolimus ointment is primarily used for steroid-resistant or intolerant OLP patients [42]. However, the local bioavailability of these conventional formulations in the dynamic, moist oral environment is typically low [16], necessitating frequent dosing by patients while efficacy remains unstable. Additionally, nystatin oral suspension is commonly used to treat oral candidiasis, but its poor taste and short contact time with the mucosa affect patient compliance and long-lasting efficacy [43]. Despite their promising potential in treating oral diseases, the therapeutic efficacy of these topical delivery systems is still limited by the three major delivery barriers inherent to oral mucosal lesions and the dynamic mechanical stress environment. Saliva flow is rapid, and muscle activities during speaking and chewing continuously mechanically disturb the application site, leading to easy dilution, washout, or enzymatic degradation of topical formulations [44]. Therefore, while topical administration has significant advantages in safety, the unique physiological environment of the oral cavity fundamentally constrains its delivery efficiency. To address these barriers, several advanced drug delivery systems have been developed. However, there is a lack of analysis regarding research trends.

To investigate the current research status and developmental trends in the field of drug delivery systems for oral mucosal diseases, this study conducted a bibliometric analysis of 303 relevant English articles published in the past five years from 2021 to 2026 based on the Web of Science Core Collection database on 9 May 2026. The retrieval formula is “oral mucosa” OR “oral ulcer” OR “oral cancer” OR “oral squamous cell carcinoma” OR “oral mucosal diseases” OR “recurrent aphthous stomatitis” (all field) and “treatment” OR “healing” OR “therapy” (all field) and “Topical Preparation” OR “Microneedle” OR “Hydrogel” OR “Oral Spray” OR “patch” OR “Film” OR “mucoadhesive polymer” OR “inject” (all field) and 2026 or 2025 or 2024 or 2023 or 2022 or 2021 (year). The analysis results showed a significant increasing trend in related research published in this field over the past five years (Figure 1a), with 2025 being the peak period for publication, and research articles being the most prevalent document type (Figure 1b). In recent years, the citation frequency of the literature related to topical delivery systems for the oral mucosa has steadily increased (Figure 1c), reflecting the sustained academic focus on overcoming the bottlenecks of traditional formulation delivery. A keyword co-occurrence analysis was performed on 265 research papers (excluding review articles) using VOSviewer 1.6.20. The minimum frequency of keyword occurrence was set to 5, after which we manually removed terms that could interfere with the analysis focusing on recent oral mucosal research, and we also unified synonymous terms for consistency. The analysis revealed that the majority of studies focused on oral ulcers and oral cancer (Figure 1d–f). In recent years, the development of oromucosal formulations has primarily centered on hydrogels, nanoparticles, films, and microneedles, with adhesion issues being the most widely researched. From a country distribution perspective, Chinese scholars rank among the top in paper output in this field (Figure 1g), indicating that domestic research teams are becoming increasingly active in the innovation and translational research of materials for oral mucosal drug delivery. Furthermore, the journals publishing the most related papers in this field include *Pharmacology and Pharmacy*, *Materials Science and Biomaterials*, *Engineering and Biomedicine*, *Nanoscience and Nanotechnology*, and *Materials Science and Multidisciplinary* (Figure 1h). This observation signifies a growing emphasis and interest in this field. Therefore, it is necessary to summarize and analyze the current publications on drug delivery systems for oral mucosal diseases. A summary and commentary on current related research will contribute to a clearer understanding of the dynamics and developmental directions in this field, providing a theoretical reference for breaking through traditional therapeutic bottlenecks and promoting clinical translation.

## 3. The Delivery Barriers of Oromucosal Formulations

Accessibility and ease of administration are two characteristics of the oral cavity. Topical oral formulations represent the most effective and convenient forms for treating oral mucosal diseases. However, many clinical trials have demonstrated that commercial formulations already fail to address therapeutic demands. These existing issues can be attributed to limited retention, accidental adhesion, treatment termination, suboptimal drug penetration, and other factors. To systematically analyze the therapeutic barriers of oromucosal formulations, we describe the oromucosal physiology and propose potential therapeutic barriers.

### 3.1. Oromucosal Anatomy

The oral mucosa can be structurally divided into three layers from exterior to interior: the epithelium, lamina propria, and submucosa (Figure 2). The epithelium is composed of multi-layered squamous epithelial cells. The basal and deeper cells retain their proliferative capacity. By virtue of differentiation and surface migration, they contribute to the multilayered structure of the epithelium [48]. According to the degree of keratinization, the epithelium includes keratinized and non-keratinized epithelia [49]. Keratinized epithelium is primarily located on the hard palate and gingiva [50]. These cells are differentiated into keratinocytes, being cuboidal or columnar in shape. Keratinocytes are tightly associated with densely distributed keratin filaments, surrounded by the cell membrane, and form the stratum corneum [51]. On the outer surface, membrane-coating granules (MCGs) begin to appear. MCGs often fuse with the cell membrane; their contents are released into the intercellular spaces to promote thickening of the cell membrane and keratin aggregation, thereby enhancing the keratinization function [51]. The keratinized epithelium demonstrates strong mechanical strength and tightly connects to the lamina propria, serving to resist external forces, decrease permeability, and reduce bacterial colonization [52]. The non-keratinized epithelium has a structure similar to the keratinized epithelium. The inside of the lips and cheeks, the floor of the mouth, and the underside of the tongue are covered by a non-keratinized epithelium [53]. Compared to the keratinized epithelium, the accumulation of keratin and MCGs in the non-keratinized epithelium is less; the increased layers of cells provide a buffer [54]. The non-keratinized epithelium plays a vital role in physiological activities such as deglutition and speech. Benefiting from MCG, non-keratinized epithelium also functions to reduce the penetration of toxic substances and the colonization of pathogens. The lamina propria and submucosa are primarily composed of connective tissue, abundant in collagen fibers, elastic fibers, blood vessels, nerves, and lymphatic vessels, providing structural support, resistance to external forces, and nutrition [48,51]. In addition, the oral epithelium is often covered by a saliva layer. Saliva is a liquid mixture produced and secreted by the salivary glands. In humans, 90% of the saliva is mainly produced by the three major pairs of parotid glands, sublingual glands, and submandibular glands. When food, odors, drugs, or external forces are present, the parotid gland will secrete a large amount of watery, amylase-rich, stimulating saliva. While in a static condition, the secreted saliva is mainly a viscous, non-irritating saliva rich in mucin, which is jointly secreted by the submandibular gland and the sublingual gland.

### 3.2. Potential Barriers for Drug Delivery to the Oral Mucosa

The unique tissue structure endows the oral mucosa with physiological characteristics that differ from other tissues. Compared to the gastrointestinal mucosa, the oral mucosa is constantly exposed to the external environment and more susceptible to various external factors such as mechanical stress, temperature fluctuations, and chemical irritants, which can induce tissue damage [55]. Meanwhile, the stratified epithelial structure significantly reduces the absorption and permeation of drugs. Compared to the epithelial structure of the skin, the oral mucosa exhibits a significantly lower degree of keratinization, resulting in more fragile structural support and protection. Furthermore, the moist oral environment and constant external exposure provide ideal conditions for the colonization and proliferation of microorganisms. When oral tissues are damaged, the complex microenvironment and ineffective drug delivery hinder tissue repair and disease recovery. Given the unique physiological characteristics of the oral mucosa, we analyzed the possible dilemma that might arise when using local oral preparations in the treatment process.

#### 3.2.1. Saliva Barrier

Saliva is a complex mixture including various components. During the analysis of the human saliva proteome, more than 3000 different proteins and peptides have been identified, including mucin, amylase, proline-rich protein, lactoferrin, immunoglobulin A, etc. In addition, various inorganic components, such as sodium, chlorine, potassium, calcium, magnesium, phosphate, bicarbonate, and trace elements, are included in saliva [56,57,58]. The complex composition is prone to triggering various physical and chemical changes in drug carriers, such as accelerated degradation, morphological changes, phase transitions, and swelling, which can affect the drug release cycle and impair therapeutic efficacy [59]. For example, oromucosal formulations based on native starch can be easily hydrolyzed by amylase, leading to a rapid drug release. This characteristic may render native starch not fit for extended-release mucoadhesive buccal formulations.

Saliva secretion continues throughout the day, with an average total secretion volume of 1–1.5 L. The amount of saliva continuously present in the oral cavity is approximately 1.1 mL [60,61]. The secreted saliva will cover all oral tissues, forming a “dynamic” hydration layer and moistening and protecting mucosal tissues [62,63,64]. According to relevant reports, the thickness of the salivary layer covering different oral tissues varies, with an average thickness of 0.1 mm [58]. Despite serving as a protector and lubricant, the saliva layer prevents the drug delivery system from closing and interacting with the mucosal tissue, which loosens the adhesion network established by the formulation and tissue, impairing drug retention at the lesion site and reducing therapeutic efficacy. When saliva secretion reaches a certain threshold, the swallowing reaction is triggered. Excess saliva on the tissue surface will be entered into the gastrointestinal tract, which is also known as saliva clearance. Saliva clearance is a common behavior in the oral cavity, playing a crucial role in removing cellular debris and bacterial attachments [58,65]. Salivary clearance involves two primary actions: continuous saliva secretion and swallowing. The flow rate of saliva depends on different physiological states. At rest, the average saliva flow rate is approximately 0.35 mL/min, while the average flow rate of saliva is approximately 2 mL/min under stimulation [60]. The continuous salivary secretion easily forms a hydration layer on the surface of the drug preparations. Due to the concentration gradients, some APIs will break away from the drug carrier and enter the hydration layer, with the help of swallowing and renewal of saliva, enter the hydration layer then the gastrointestinal tract. This process reduces the drug concentration at the lesion site. In addition, saliva clearance renews the “dynamic” saliva layer on the tissues’ surface. With the renewal and flow of the salivary layer, the fragile adhesion network enters the “broken-rebuild” cycle. These behaviors further aggravate the loss of adhesion, which also explains why some materials and formulations have good performance in in vitro adhesion experiments, but exhibit detachment and poor adhesion in in vivo environments.

Overall, saliva is the first obstacle encountered by oromucosal formulations. Saliva not only causes structural and functional damage to drug delivery systems but also leads to drug leakage, reducing therapeutic efficacy and triggering potential adverse reactions.

#### 3.2.2. Gel and Epithelial Barrier

The oral mucosa is the unique soft tissue in the oral environment, involved in various oral movements such as chewing, speaking, and swallowing. The lubricated oral mucosa also makes an important contribution to the oral moist environment. However, there are two delivery obstacles on the oral mucosa: limited adhesion and permeation.

##### Limited Adhesion

The surface of the oral mucosa is covered by a gel layer composed of multiple components including mucins, immunoglobulin A, proline-rich proteins, and inorganic ions, which are formed through various interactions such as hydrophobic forces and metal chelation [64,66]. The gel layer exhibits relatively low friction, which can be attributed to the presence of mucins MUC1 and MUC5B [67]. Extensive studies confirm that MUC1 and MUC5B significantly decrease the friction and enhance lubrication function in the oral cavity [68,69,70,71]. The lubricating capacity of mucin is mainly derived from the following two points: (1) The mucin molecule is composed of a densely glycosylated, hydrophilic core flanked by two hydrophobic termini. The glycosylated core of the mucin molecule contains negatively charged sialic acid residues and sulfated glycans, which are used to capture water molecules. Under the action of mucin, water molecules are closely arranged and form a dense hydration layer. The rapid relaxation dynamics induce the hydration layer to exhibit fluid-like behavior and reduce friction under external shear force [72,73]. Research has shown that when the polysaccharides of the mucin are removed, the water interaction and friction coefficient can decrease 3.5-fold and increase by two orders of magnitude, respectively [74]. (2) Hydrophobic interactions expose the hydrophilic groups of mucin to the external environment, thereby intensifying the intermolecular repulsion. The brush-like and fibrous structure appears due to the steric repulsion between mucin molecules. The spatial steric hindrance and extended surface layer prevent the relative surfaces from penetrating, reducing the adhesion area between the drug carrier and oral tissue, and thus reducing the adhesion strength [72]. Therefore, breaking the interference of mucin or interacting with mucin may be an important method to increase the retention time.

##### Limited Permeation

Poor drug permeability is fatal because it leads to insufficient drug concentration at the lesion site and unsatisfactory therapeutic effects. From the anatomical structure of the oral mucosa, the limited permeation mainly involves the mucus layer and the epithelial layer.

The mucus barrier refers to the obstruction of the gel layer. As mentioned earlier, the gel layer is a network formed by the interaction of various proteins. The network will impede the delivery of the drug to its destination when it is passing through the gel layer. This resistance can be divided into two aspects: size filtration and physical–chemical interactions [75,76]. When the drug particle size is larger than the mesh size, the drug molecules will be retained and remain in the gel layer, which is called size filtration. Research by Goswami et al. indicates that the pore diameters for the absorption of hydrophilic compounds through the buccal mucosa and sublingual mucosa should be lower than 22 Å and 53 Å [77]. For most small-molecule drugs, the size of the reticular structure is at least 100 times larger than most common drug molecules [76]. However, for biomacromolecules, the restriction of the mesh is a headache for scientists. Different from mesh restriction, physical–chemical interaction restriction seems to affect any drug. Gargano et al. evaluated the retention situation of different drugs through mucin fixed phase, and the results showed that mucin has a strong binding force for a variety of small molecule drugs [78]. Lieleg et al. indicate that particle migration in the gel layer is related to their surface charge [79]. Higher surface potential exhibits a stronger inhibition. Similarly, Falavigna et al. reported that the presence of mucin significantly reduces drug permeability, which is concentration-dependent [80]. These results suggest that mucin may affect drug permeability through multiple interactions.

After crossing the mucosal barrier, the drugs accumulate on the surface of the mucosal epithelium. On the mucosal epithelium surface, the drugs can be transported through transcellular pathways and paracellular pathways. However, various evidence indicates that the outer one-third to one-fourth area of the mucosal epithelium may be a barrier for drug transport. Marxen et al. used mass spectrometry imaging technology to analyze the deposition of different drugs on the oral mucosa, and the results showed that the vast majority of the drugs were located in the outer quarter of the epithelium [81]. Squier’s research pointed out that after separating the mucosal epithelium, the permeability of the drugs increased by 2–8 times [82]. Additionally, many researchers also reached the same conclusion [83,84,85,86]. It can be seen that the presence of the mucosal epithelium seriously hinders the penetration and absorption of drugs.

Based on these findings, scientists have been exploring the reasons why the mucosal epithelium hinders the diffusion of drugs. Firstly, the properties of the drugs and carriers. The ionization state, lipophilicity, molecular weight, and even the groups on the drug molecule can all affect the efficiency of drug penetration. Goswami et al. pointed out that the lipophilicity of the drug has a linear relationship with drug penetration, and ionization contributes more significantly to drug penetration [87]. In another research study, Wanasathop et al. established and analyzed a permeability parameter database, deriving an effective pore radius of 1.5 to 3 nm for drug permeation through the oral mucosa [88]. Moreover, the presence of excipients also exerts a certain influence on the drug’s permeability. For example, Jeitler et al. investigated the interaction between lipid nanoparticles and oral epithelial cells, concluding that oleic acid will enhance epithelial cellular uptake of the lipid nanoparticles [89]. Secondly, the components of the epithelial layer. The oral epithelium is composed of stratified squamous epithelial cells. The complex structure and components are major obstacles to drug transportation. Law et al. investigated the lipid content and distribution of the oral epithelium, revealing that ceramides may be a key component impeding drug penetration [90,91,92,93]. Imafuku and colleagues analyzed the distribution and appearance of ZO-1 protein in buccal mucosa and demonstrated its barrier function against small molecule permeation [94]. The oral epithelial layer also contains many MCGs composed of polar lipids, which release their contents into the intercellular space to form a lipid barrier, hindering the penetration of compounds [91,95,96].

#### 3.2.3. Oral Complex Biomechanical Environment

During the development of oral formulations, scientists have focused on drug adhesion and penetration. The dynamic oral environment is often overlooked. Mandibular movement causes rapid and repetitive stretching and retraction of buccal mucosa during mastication [97,98]. This stretching and retraction will disrupt the physical structure of pharmaceutical preparations and alter formulation morphology, thereby affecting drug absorption. Formulations with greater hardness (such as freeze-dried patches) tend to cause secondary injury to wounds and nearby tissues. Compared to the “static” oral cavity, the “dynamic” tongue can reach every region within the oral cavity. Research indicates that tongue muscle stress of 12-year-old adolescents reaches up to 62 kPa [99]. The foreign body sensation from patches may induce involuntary tongue “aggression,” subsequently causing accidental adhesion and structural damage. Oral tissue movement disrupts the network structure and appearance of formulations, resulting in periodic drug release irregularity. Thus, oral formulation design must fully account for the dynamic oral environment.

### 3.3. Summary

The oral environment is characterized by complexity, dynamics, and wetness, which poses significant challenges for the design of oral preparations. Although oral formulations demonstrate excellent compliance, there are numerous factors needed to be considered. The adhesion and permeation barriers, the deformation of the mucosa and the mechanical stress of the tongue, the complex composition and secretion volume of saliva, etc., are all factors that we need to take into careful consideration. We need to consider the various performance aspects of the preparations, rather than just a single element. This might pose severe challenges for the design of the preparations.

## 4. Design Strategies for Oromucosal Formulations

Based on the above analysis, the challenges in oral drug delivery primarily manifest in three aspects: insufficient adhesion strength of formulations, poor drug permeability, and inadequate formulation performance. To address these challenges, scientists have proposed a range of strategies. Table 4 summarizes the various strategies and specific measures.

### 4.1. Strategies for Enhancing Adhesion

#### 4.1.1. Eliminating the Interference of Saliva

The presence of the salivary layer poses challenges to the adhesion properties of drug delivery systems. Therefore, it is essential for the adhesion of pharmaceutical preparations to remove the salivary layer. To address this challenge, two methods for managing interfacial water have emerged in the field of biomedicine: absorbing and removing interfacial water, respectively. As previously reported, dry formulations effectively absorb interfacial water and adhere in situ when contacted with wet tissues. Such formulations include dry gels, films, powders, etc. Simplified manufacture is a strength of dry formulations, which reduces the cost of production. The majority of dry formulations are prepared via drying the precursor solution, where water is the primary solvent to ensure safety. Freeze drying, vacuum drying, and even air drying are commonly used dehydration processes. The straightforward dehydration process makes dry formulations popular in laboratory R&D, industrial production, and clinical applications. In addition, rapid adhesion is another notable advantage. Compared to liquid and semi-solid formulations, dry formulations can rapidly absorb water to achieve strong adhesion. Nowadays, the interfacial water absorption strategy is widely applied in severe hemorrhage control, gastrointestinal perforations and ulcers, wound infection, and more [101,121,122,123].

With further research into oral formulations, scientists discovered that dry formulations are also suitable for topical oral diseases. For example, Wang et al. engineered a dry gel patch (PACA) [100] (Figure 3a). The PACA exhibits excellent wet adhesion, maintaining over 30 kPa adhesion strength to oral mucosa soaked in saliva (Figure 3b). In the hamster buccal pouch carcinogenesis model, PACA significantly inhibited tumor proliferation, with the treated tumor area being only one-seventh that of the conventional treatment group (Figure 3c). Self-gelling powders are also commonly used for oral tissue adhesion. When sprayed onto tissue surfaces, these powders rapidly absorb saliva and form multiple interactions with tissue through exposing adhesive groups [101,122,123]. Liu et al. designed a patient-friendly sprayable rapid gelation powder (SRGP) for oral ulcer therapy [101] (Figure 3d). Fluorescence results showed that the hydrogel maintained approximately 25% coverage of the wound area after 12 h (Figure 3e,f). The strong adhesion of the hydrogel was further demonstrated by its stability under torsion, stretching, and water jet conditions. These experimental results prove that SRGP is capable of serving as a drug delivery system for the oral cavity. Film is commonly used as a dry formulation and widely adopted due to simple manufacture. For instance, Liao et al. prepared freeze-dried films with dopamine (DA) and chitosan (CS) as raw materials, achieving in vivo adhesion for up to 2 h [102]. Ge and Racaniello et al. separately developed drug-loaded films using electrospinning and 3D printing technologies, obtaining excellent performance and positive therapeutic effects [124,125]. The incorporation of hydrophobic materials into polymer matrices appears to be a promising approach, which is also described as a strategy to exclude the hydration layer. The low water absorption characteristic prevents the formulation structure from excessive swelling and damage while maintaining stable adhesion. The sustained adhesive effect also benefits from the stable physical structure of the formulation. Gosecka and colleagues elucidated several strategies for constructing hydrophobic hydrogels in their research, including micelle formation, embedding of micron/nanoparticle systems, and biphasic gels [126]. These strategies offer significant inspiration for the engineering of wet adhesive hydrogels. For example, polycaprolactone triol (CAPA3091) and 2,2-dihydroxymethylpropionic acid (DMPA) were used to modify waterborne polyurethane, producing the adhesive patch CDCPU in Guo’s research [127]. CDCPU exhibits durable adhesion and resistance to hydration. As shown in Figure 3g, the shear-lap and tensile adhesion strength of the CDCPU patch to porcine oral mucosa is 12.0 kPa and 15.7 kPa, significantly higher than commercial ulcer membranes (<10 kPa). The addition of hydrophobic precursors reduces the water absorption of the patch (Figure 3h). Similarly, Du et al. constructed a hydrophobic hydrogel PVA-DOPG via polyvinyl alcohol (PVA) and dioleoyl phosphatidylglycerol (DOPG) [128]. Characterized by high adhesion and permeability, PVA-DOPG effectively decreased the incidence of OSCC.

Two primary strategies for interfacial water management have drawn attention for oromucosal drug delivery. However, numerous new challenges have emerged during the development of oral formulations and have become significant obstacles. (1) Continuous salivary absorption greatly increases swelling, disrupting the formulation structure and leading to disintegration. (2) Although hydrophobic materials can delay salivary erosion, complex purification processes may pose potential biosafety risks. (3) The carrier is often required to establish a safe and bioactive environment, which is particularly important for macromolecular drugs. The anhydrous environment in dry formulations and the long-chain groups introduced by hydrophobic strategies may impose higher demands on drug stability.

**Figure 3:**
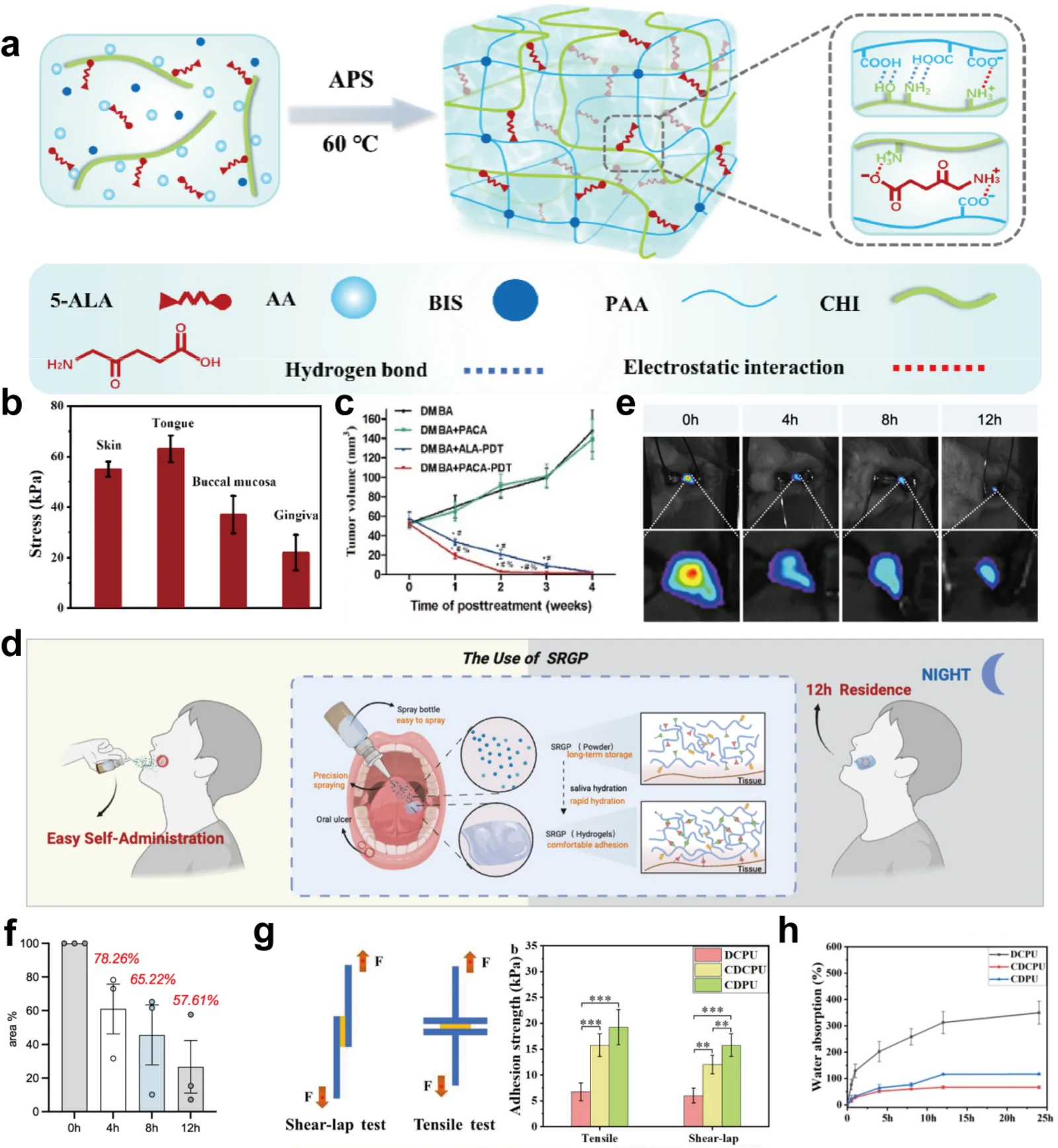
Oromucosal drug delivery system based on saliva evaporation. (**a**) The preparation process for PACA dry gel patches. (**b**) Shear strength of PACA patches on porcine skin, tongue, buccal mucosa, and gums soaked in saliva. (**c**) The tumor volume–change curves for different treatment groups after four weekly treatments. * *p* < 0.05 compared to the DMBA group; # *p* < 0.05 compared to the DMBA + PACA group; % *p* < 0.05 compared to the DMBA + ALA-PDT group. (**d**) A diagram illustrating the application of SRGP and the gelation process within the oral environment. (**e**) Fluorescence retention of SRGP in the rat oral cavity at different time points. (**f**) The quantitative analysis of fluorescence retention. (**g**) The left side shows a schematic diagram of the shear-lap and tensile tests, while the right side shows the shear-lap strength and tensile adhesion strength of different formulations in contact with porcine oral mucosa after 24 h. Quantitative data were all expressed as mean ± SD, and tests were performed at least in triplicate. Statistical analysis between data was performed by one-way analysis of variance (ANOVA) using GraphPad Prism software, and *p* values < 0.05 were considered to be significant (** *p* < 0.01, *** *p* < 0.001). (**h**) Water absorption of different formulations at different times. Panels (**a**–**c**), panels (**d**–**f**) and panels (**g**,**h**) adapted with permission from Refs. [100,101,127]. Copyright © Adapted from Elsevier.

#### 4.1.2. Enhancing the Interaction with the Tissue and Mucus Layer

Multiple proteins are distributed across the oral mucosal surface, which contain various functional groups (primary amines, carboxyl groups, hydroxyl groups, thiols, etc.), constituting the adhesion sites in the oral mucosa. Compatible polymers can be utilized to form diverse interaction forces with oral mucosa, such as hydrogen bonds, dynamic covalent bonds, and electrostatic interactions, significantly enhancing the adhesion strength and time of formulations at the oral mucosa [59,129]. However, relying on a single adhesion mechanism often yields suboptimal results; synergistic effects of multiple adhesion forces are required to achieve effective adhesion. Furthermore, complex polymer structures tend to establish multiple interaction forces with tissues. Therefore, this review primarily introduces representative adhesion systems applied in oral mucosa.

##### Non-Covalent Interaction

Noncovalent interactions refer to intermolecular physical forces that are ubiquitous in nature. Common physical interactions such as hydrogen bonding, electrostatic interactions, van der Waals forces, and cation–π interactions serve as the foundation for oral adhesive formulations.

Electrostatic interaction is one of the fundamental adhesion mechanisms. It involves competition between the polymer and the hydration layer for binding to mucin. The positive charge of the polymer attracts the negative groups on mucin, thereby preventing the hydration layer from forming and enhancing adhesion. Pham et al. further identified this interaction as potentially resulting from binding to the N-acetyl groups on the mucin glycan side chains and polypeptide chains [130]. This can be considered a specific recognition mechanism. In their work, positively charged PEI was shown to interact with fragments at 3.5–4.5 ppm and may alter the conformation of mucin, increasing the mobility of some N-acetyl groups. This specific recognition distinguishes it from uncharged and negatively charged polymers. Common cationic polymer materials include chitosan, polyethylenimine (PEI), polylysine, and guar gum. Among these, chitosan is the most widely used material. For example, Zheng et al. constructed a triamcinolone acetonide-loaded hydrogel patch (CFT) using fucoidan-grafted chitosan as the carrier [131] (Figure 4a). The prepared hydrogel patch exhibited the capability to bear up to 50g and adhesion strength higher than 40 kPa (Figure 4b,c). Ni and Qi et al. engineered injectable chitosan gels for oral mucosal wound healing studies [132,133]. Thanks to the mucosal adhesion properties of chitosan and the high contact interface of injectable gel, all final formulation groups achieved favorable therapeutic efficacy. In a Phase II clinical trial titled “α-Mangostin Hydrogel Film With Chitosan Alginate Base for Recurrent Aphthous Stomatitis (RAS) (AM-RAS)” (ClinicalTrials.gov ID: NCT06039774, Study Registration Dates: 14 September 2023), the preclinical studies conducted on white Wistar rats demonstrated a remarkable ulcer healing rate of 93.0% within 7 days [134]. This trial is currently underway to advance clinical and safety evaluations. Taken together, these findings indicate that chitosan, as a material for enhanced adhesion, has achieved a considerable level of maturity and holds promising potential for clinical applicability.

Hydrogen bonds are the most prevalent noncovalent interactions. The diverse amino, hydroxyl, and carboxyl groups in oral mucosa provide abundant electron-deficient hydrogen atoms, laying the foundation for hydrogen bond formation. While individual hydrogen bonds are relatively weak, multiple bonds can yield a significant adhesive effect [135]. Polymers based on hydrogen bonding interactions can be categorized into neutral polymers (such as polyvinyl alcohol, polyethylene glycol, cellulose derivatives, and polyvinylpyrrolidone) and anionic polymers (such as polyacrylic acid, sodium alginate, and hyaluronic acid). The reversibility allows hydrogen bonds to maintain a dynamic “adhesion-release” process with tissues. This unique mechanism enables hydrogen-bonded polymers to effectively adapt to the dynamic oral environment and achieve excellent adhesion. Liu et al. fabricated hyaluronic acid soluble microneedle patches (HEMC MNs) for accelerating oral ulcer healing [136]. Capitalizing on the multiple hydrogen bonds of HA, HEMC MNs can adhere stably on the mucosal surface under mild pressure. As shown in Figure 4d, the adhesion strength of HEMC MN patches was approximately 1.6 times that of the commercial product. The same conclusion was also supported by other scientific researchers. Alhallak et al. noted that hydrogen-bonded polymer films exhibit prolonged retention time on oral mucosa (>24 h) and satisfactory adhesion ranges (2.72N–4.03N) (Figure 4e) [105]. Another study by Zhou et al. demonstrated that a polyacrylamide/sodium alginate crosslinked hydrogel maintained adhesion to wet interfaces for up to 24 h with a tensile force of 1N (Figure 4f,g) [137].

**Figure 4:**
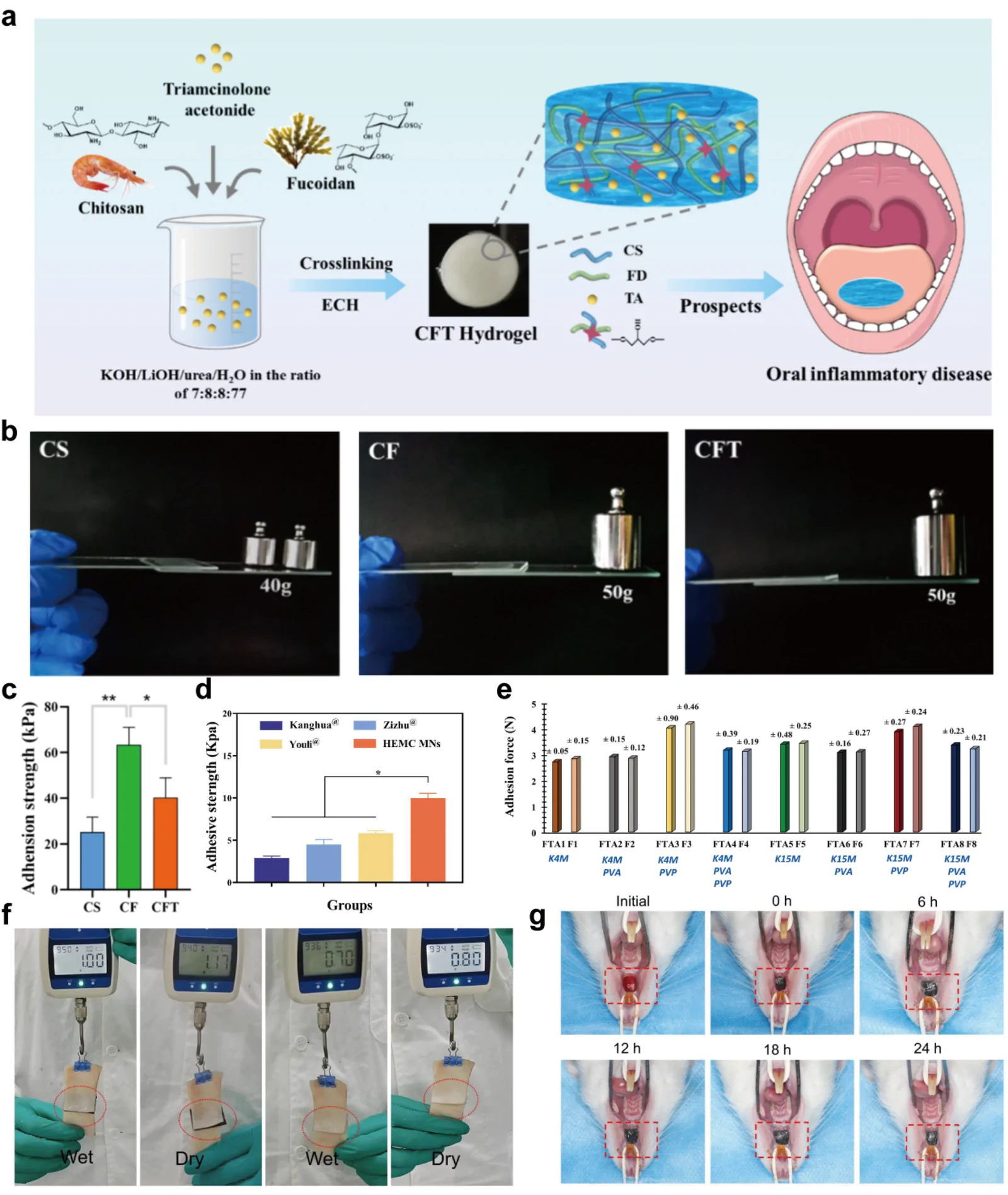
Oromucosal drug delivery system based on noncovalent interactions. (**a**) Schematic diagram of the CFT hydrogel preparation process and its applications in oral diseases. (**b**) Photographs of in vitro adhesion for different hydrogels. (**c**) Comparison of shear adhesion strength among CS, CF, and CFT hydrogel films. *p* values < 0.05 were considered to be significant (* *p* < 0.05, ** *p* < 0.01). (**d**) Comparison of adhesion strength between HEMC MNs and other commercial buccal films (Kanghua film^®^, Zizhu film^®^, and Youli film^®^). Panels (**a**–**c**) and Panel (**d**) adapted with permission from Refs. [131,136]. Copyright © Adapted from Elsevier and Springer Nature. (**e**) Comparison of the adhesion force to buccal mucosa among adhesive films with different formulations. FTA: drug-loaded film, F: drug-free film. Panel (**e**) adapted with permission from Ref. [105]. Copyright © Adapted from Elsevier. (**f**) Comparison of the adhesive forces of PAA-SA and PAA-SA@rGO hydrogels on dry and wet porcine skin. (**g**) The adhesion retention time of PAA-SA@rGO hydrogels in the oral cavity. Panels (**f**,**g**) adapted with permission from Ref. [137]. Copyright © Adapted from John Wiley and Sons.

##### Covalent Crosslinking System

Covalent crosslinking refers to the process where adhesive groups of polymers and reactive groups on tissue surfaces form chemical crosslinks and bonds. The bond energy of covalent bonds is higher than that of physical interactions, indicating stronger adhesion effects. Covalent crosslinking systems include Schiff base, amide bonds, disulfide bonds, borate bonds, etc.

Schiff bases are formed through the condensation reaction between primary amines (-NH_2_) and carbonyl groups (-C=O) or aldehyde/ketone groups. This reaction is characterized by rapidity, reversibility, immediacy, and strong adhesion. Schiff base is deemed to play a role in adhesive networks. Numerous reports indicate that Schiff base adhesive networks are frequently utilized as wound dressings and tissue adhesives. It is essential for constructing rapid and highly adhesive Schiff base networks to increase the aldehyde content. Polysaccharide oxidation represents the most traditional approach. Such methods often employ periodate as an oxidant to cleave ortho-diols along polymer chains and generate adjacent aldehyde groups. Common examples include oxidized dextran, oxidized hyaluronic acid, and oxidized Pluronic F-127, etc. For instance, Ni et al. constructed an injectable hydrogel (SQD1) based on dialdehyde scleroglucan and quaternized chitosan (Figure 5a) [132]. When applied to ulcers, the free aldehyde reacts with tissue and chitosan, resulting in gel formation and adhesion to the wound. After 6 days of treatment, the ulcer area was significantly reduced (Figure 5b). Similarly, Yang et al. engineered an injectable hydrogel composed of oxidized cellulose and chitosan for repairing soft-tissue defects [138]. Following 10 days of treatment, the wound area was nearly fully healed. Aldehyde grafting also appears to be a feasible scheme. Wang et al. grafted aminoacetaldehyde dimethyl acetal with HA (aHA), which is blended with quaternized chitosan, and pyrocatechol aldehyde to synthesize the bioadhesive AHP [139]. Due to the effect of the aldehyde group, AHP can stably adhere to stretched or twisted pig skin (Figure 5c). However, adhesion depends on the aldehyde content reacted with tissue. Premature generation of aldehydes may lead to excessive oxidation and consumption. Therefore, to improve the competitiveness of tissue amino, phototriggered aldehyde production offers a fair opportunity. Generally, 2-Nitrobenzyl alcohol is grafted into polymer chains. Under exposure to ultraviolet light, 2-Nitrobenzyl alcohol transforms into 2-Nitrosobenzaldehyde (NB). The generated aldehyde group can form Schiff bases with amino groups in tissues, achieving tight contact. Several studies have reported this technical route. Xiang et al. coupled nitrobenzyl-modified chondroitin sulfate (CHS-NB) with methacrylate silk fibroin to synthesize photoresponsive hydrogels (SCE2). After being injected, SCE2 fits closely to the wound; then, the gelation and adhesion process occurs upon UV irradiation (Figure 5d) [23]. Zhang et al. developed a cyclic o-nitrobenzyl-modified hyaluronic acid gel (HA-CNB) for protecting mucosal wounds [140]. SEM imaging revealed tight tissue interface contact with HA-CNB (Figure 5e). Even the weakest HA-CNB gel exhibited adhesion strength exceeding 40 kPa (Figure 5f). Fluorescence results from mucosal wounds further demonstrated the hydrogel’s retention in the rat oral cavity for up to 24 h (Figure 5g). Chen et al. found that the adhesion strength of the hydrogel increased with NB concentration (Figure 5h) [141].

The amide bond is an irreversible covalent bond whose adhesive strength significantly surpasses that of other chemical bonds. The formation of amide bonds involves interactions between amino groups on tissue surfaces and carboxyl groups on polymer chains. However, their condensation reaction often cannot proceed spontaneously. Only after the carboxyl group is activated and a higher bond energy is given can it react with the amino group. N-Hydroxysuccinimide (NHS) is a carboxyl group activator that can generate the R-COO-NHS structure. Then, R-COO-NHS can be replaced with an amide bond. This reaction proceeds relatively rapidly, with reaction times ranging from seconds to minutes. Rapid, strong, and durable adhesion are the characteristics of the NHS adhesive system [135]. Zhang et al. designed a sprayable hydrogel (LP) for oral ulcer treatment, composed of lysozyme (LZM) and 4-arm-PEG-NHS [103] (Figure 5i). Even after repeated water shearing (up to 2000 cycles), LP maintains structural integrity (Figure 5j). Zhang and colleagues developed the adhesive hydrogel patch ASNB containing gelatin, acrylic acid, and acryloyl NHS ester [118]. The adhesion strength of ASNB exceeded 40 kPa in all the adhesive tests (Figure 5k). Even after 48 h of immersion in water, ASNB maintains an adhesion strength of 15 kPa (Figure 5l).

Abundant cysteine subdomains of mucin provide binding targets for polymers. These subdomains form disulfide bonds with thiol-containing polymers, enhancing adhesion. When in contact with the oral cavity, thiolated polymers permeate the gel layer, effectively settling in the mucin network and forming stable disulfide bonds [142,143,144]. Consequently, thiolated polymers are regarded as promising mucosal adhesion materials. For instance, Grewal et al. prepared thiol-modified Moringa gum oral patches (TMG) [106]. As shown in Figure 5m, TMG exhibits a separation force from mucosal surfaces up to 1070 mN, surpassing that of moringa gum. Ebrahimnia et al. synthesized thiolated chitosan and gelatin to construct nanofibers [145]. In vitro adhesion experiments demonstrated that the mucoadhesion of thiolated nanofibers showed an average of 432.7 Pa, which was significantly higher than that of the non-thiolated nanofibers (about 5 times) (Figure 5n). Otherwise, in another work by Meenakshi Bhatia, thiol modification extended the adhesion time of xanthan gum by five-fold [146].

**Figure 5:**
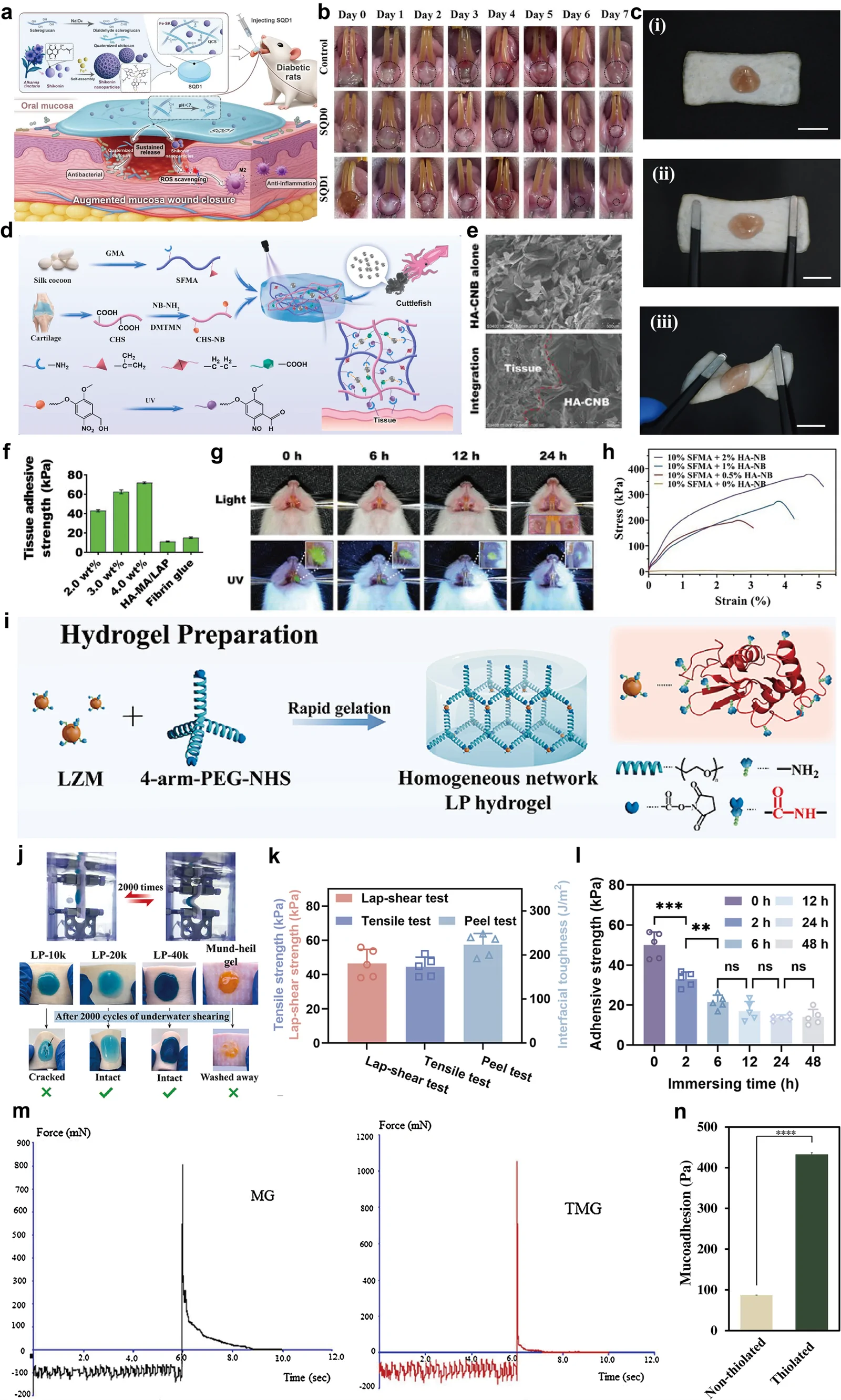
Oromucosal drug delivery system based on covalent interactions. (**a**) Preparation diagram of SQD1 and its application in the treatment of oromucosal wounds in diabetic conditions. (**b**) Ulcer healing progression over 7 days in the SQD0, SQD1, and control groups (the black dashed box indicates the ulcer site). (**c**) The AHP adhesive test on pig skin under stretched and twisted conditions. (i) Resting state; (ii) Tensile state; (iii) Twisted state. Scale: 1 cm. (**d**) Schematic illustration of the synthesis of SCE2 hydrogel and its adhesion to tissue under UV irradiation. Panels (**a**–**d**) adapted with permission from Refs. [23,132,139]. Copyright © Adapted from Elsevier. (**e**) SEM observation of the integration of the HA-CNB gel with rat muscle tissue (red dashed line indicates the boundary). (**f**) The adhesion strength of different hydrogels to hog casing was evaluated using a standard lap shear test. (**g**) The retention of fluorescein-labeled HA-CNB in the rat oral cavity. (**h**) Tensile-adhesion curves of hydrogels at different HA-NB concentrations. Panels (**e**–**h**) adapted with permission from Refs. [140,141]. Copyright © Adapted from John Wiley and Sons. (**i**) Schematic diagram of LP hydrogel preparation. (**j**) Retention performance of LP hydrogel with different molecular weights and Mund-Heil gel after 2000 cycles of underwater shearing. (**k**) The adhesive strength of ANSB in lap shear, 180-degree peel, and tensile tests. (**l**) Variation in adhesion strength in hydrogels after different immersion times in artificial saliva. *p* values < 0.05 were considered to be significant (** *p* < 0.01; *** *p* < 0.001; ns, not significant). (**m**) The force-time curve of Moringa gum and thiolated Moringa gum compacts. (**n**) Mucoadhesion of thiolated and non-thiolated nanofibers. *p* values < 0.05 were considered to be significant (**** *p* < 0.0001). Panels (**i**–**k**,**m**,**n**) adapted with permission from Refs. [103,106,118,145]. Copyright © Adapted from Elsevier.

##### Multiple Synergistic Interactions

Oral mucosa is a sophisticated tissue with diverse adhesion groups, providing numerous binding sites. The development of oral formulations faces significant challenges. Rapid adhesion capability requires oral formulations to quickly eliminate interfacial saliva and achieve close contact with the tissue. Sustained adhesion time depends on the robust and stable covalent networks between the formulation and tissue, resisting saliva washout and mechanical shear stress. Furthermore, to fulfill patient needs, an oral patch is often required for its detachable adhesive capability to prevent accidental administration to healthy tissues. A single adhesive mechanism struggles to fully resolve these challenges. Consequently, formulations with multiple tissue adhesion capabilities have emerged as an emerging clinical requirement.

α-Lipoic acid, a sulfur-containing five-membered cyclic compound, features a core structure of 1,2-dithiolane-3-propanoic acid. Upon exposure to light or elevated temperatures, the disulfide bond cleaves homolytically and facilitates ring-opening polymerization to form poly(lipoic acid)(PolyLA) [147] (Figure 6a). PolyLA is a hydrophobic polymer rich in carboxyl groups. Its remarkable adhesive properties are attributed to hydrogen bonding, electrostatic interactions, and hydrophobic interactions. Inspired by research on α-lipoic acid, Liu Wenguang’s research group has developed several characteristic α-lipoic acid formulations for oral ulcer management. In early 2023, Liu Wenguang and colleagues integrated water-soluble PolyLA-Na into the PolyLA network, constructing an oral patch (PolyLA-Na/PolyLA) [25]. When the oral patch comes into contact with the oral mucosa, the interfacial water will be absorbed, and the PolyLA will be released to generate an adhesive network. The result showed that the highest instant adhesion strength was up to 60 kPa (Figure 6b). Two years later, the authors incorporated sodium queen bee acid (SQBA) into the PolyLA to develop the PolyLA-SQBA patch [148]. With the self-assembling micelle properties, PolyLA is temporarily encapsulated within the SQBA micelles. When the temperature rises to 37 °C, the PolyLA is released from the micelles, generating adhesion (Figure 6c). This thermosensitive patch exhibits adhesion fault tolerance for in vivo application. Leveraging the “tea polyphenols + alpha-lipoic acid” combination, the authors also developed an oral patch (PLTP) for regulating iron metabolism imbalances, achieving positive results (Figure 6d) [33]. Otherwise, Chen et al. designed an on-demand peelable lipoic acid Janus patch [149]. When a GSH/NaHCO_3_ solution is applied, the patch can be rapidly peeled off, with adhesive strength sharply decreasing within 15 min (Figure 6e).

Many organisms have evolved unique wet-surface adhesion strategies over long periods of evolution, contributing to numerous remarkable wet-surface adhesion phenomena that serve as inspiration for scientists to design wet adhesives. Biomimetic adhesives are a kind of polymer derived from or inspired by nature. Mussel-inspired adhesive polymers are the most widely applied polymers in the oral cavity. Mussels have abundant 3,4-dihydroxy-L-phenylalanine (DOPA), tyrosine, phenylalanine, and various charged groups [150]. Numerous studies indicate these residues confer wet adhesion through hydrophobic interactions, cation–π interactions, or electrostatic interactions, etc. [151]. DOPA is considered the most crucial amino acid for wet adhesion. Studies demonstrate that the catechol-containing peptide in DOPA can penetrate the water boundary layer and form interfacial bonds with underwater substrates [152,153]. Inspired by mussels, scientists have developed many methods to incorporate DOPA and its derivatives into polymers, including molecular chain grafting, functional module addition, and monomer incorporation. Choi et al. grafted 5′-hydroxytryptamine (PG) onto pectin chains (Pec) to prepare a self-adhesive in situ hydrogel (Figure 6f) [154]. Even after being repeatedly washed with PBS, Pec-PG retained noticeable fluorescence on the tongue (Figure 6g). The enhanced adhesion is likely attributed to multiple interactions such as disulfide bonds, amide bonds, and electrostatic interactions, which are driven by the semiquinones and phenoxy radicals (Figure 6h,i). Similarly, Hu et al. grafted DOPA onto PVA chains, yielding PVA-DOPA polymers with adhesion strengths up to 40 kPa [155]. Li and colleagues took the tannic acid monomers and tannate-functionalized nanoparticles into the PVA solution to prepare the oral patch PT@Zn for the OSCC treatment (Figure 6j) [156]. As shown in Figure 6k, PT exhibited significantly higher shear strength and interfacial toughness than pure PVA. Thanks to excellent adhesion, the PT@Zn patch adhered to the oral mucosa of SD rats for over 36 h (Figure 6l). Li et al. developed an injectable hydrogel (DCE1) by integrating xanthan gum, chitosan, and EGCG nanoparticles [157]. In the tensile test, DCE0 exhibited an adhesion strength of 35.9 kPa, while DCE1 reached 44.8 kPa. This enhanced tissue adhesion primarily resulted from the formation of multiple interactions between EGCG nanoparticles and tissues.

**Figure 6:**
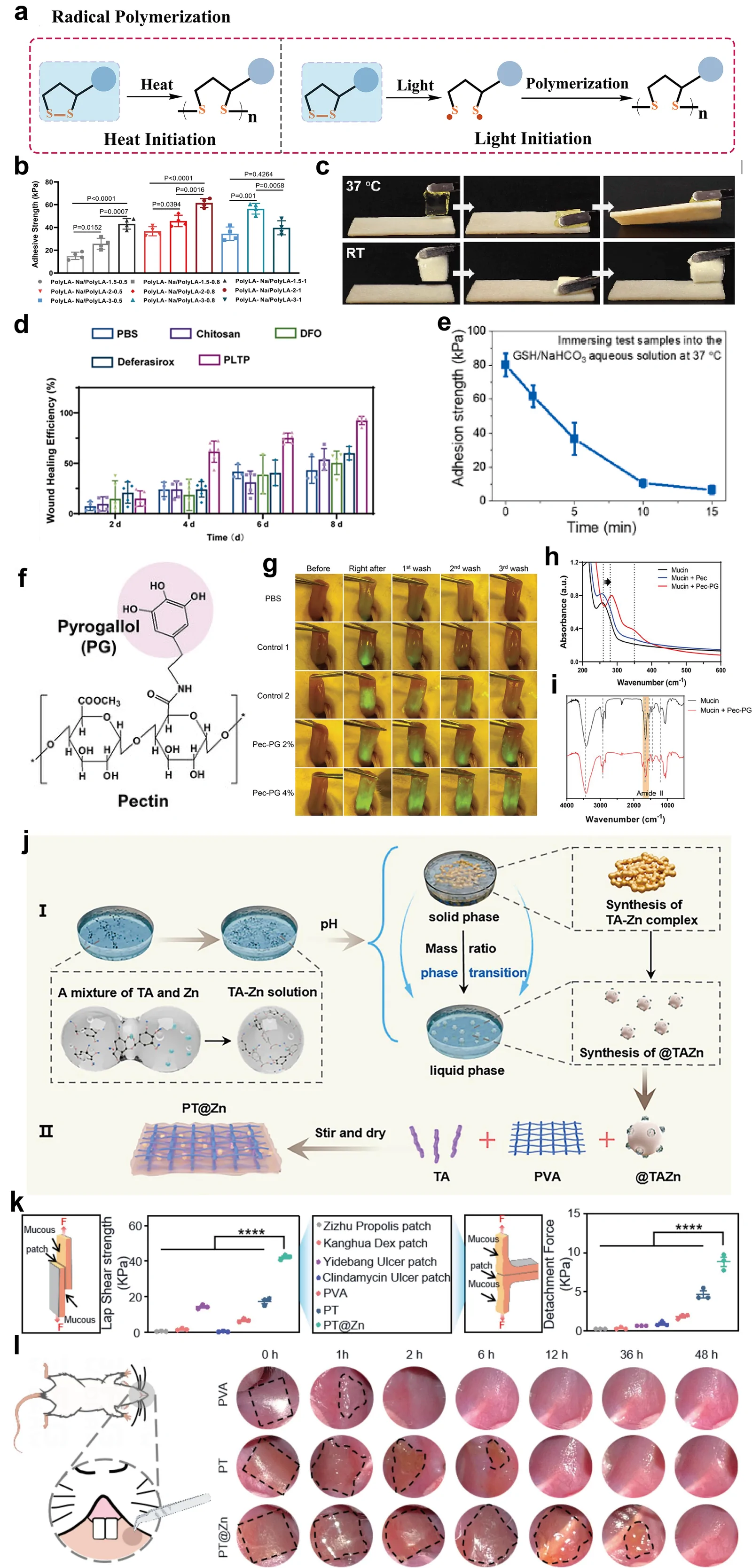
Oromucosal drug delivery system based on multiple synergistic interactions. (**a**) Schematic diagram of the ring-opening polymerization of α-lipoic acid under heat and light initiation. Panel (**a**) adapted with permission from Ref. [147]. Copyright © Adapted from Elsevier. (**b**) Adhesion strength of PolyLA-Na/PolyLA patches with different compositions to the oral mucosa after immersion in artificial saliva. Panel (**b**) adapted with permission from Ref. [25]. Copyright © Adapted from John Wiley and Sons. (**c**) Photo of PolyLA-SQBA-30-20 patch thermal response adhesion to pigskin. (**d**) Oral wound healing efficiency of rats receiving different treatments at various time points. (**e**) Changes in the adhesion strength of Janus films over time following immersion in GSH/NaHCO_3_ solution at 37 °C. Panels (**c**–**e**) adapted with permission from Refs. [33,148,149]. Copyright © Adapted from Elsevier. (**f**) Chemical structure of Pec-PG. (**g**) After spraying onto rat tongues and washing with PBS, fluorescence remaining of fluorescein isothiocyanate (FITC)-labeled Pec-PG hydrogels and commercial control agents. (**h**) UV absorbance spectra of mucin, mucin + Pec, and mucin + Pec-PG. (**i**) FTIR spectra of mucin and mucin + Pec-PG. Panels (**f**–**i**) adapted with permission from Ref. [154]. Copyright © Adapted from John Wiley and Sons. (**j**) Synthesis route of zinc tannate nanoparticles (@TAZn) and manufacturing for PT@Zn oral patches. I. Zinc tannate nanoparticles were synthesized by phase regulation. II. Schematic of PT@Zn oral patch synthesis. (**k**) Shear strength and interfacial toughness of PVA, PT, PT@Zn and various commercial oral patches. *p* values < 0.05 were considered to be significant (**** *p* < 0.0001). (**l**) Schematic diagrams of the adhesion of oral patches to the oral mucosa, as well as their in vivo adhesion and degradation times. Panels (**j**–**l**) adapted with permission from Ref. [156]. Copyright © Adapted from Elsevier.

#### 4.1.3. Summary

The moist oral interfacial environment poses a formidable technical obstacle to the development of topical drug delivery formulations. To overcome the challenges presented by such wet interfaces, it is necessary to systematically design formulation systems with tailored properties from multiple perspectives. Based on a systematic review of recent advances, we consider that enhancing the surface dryness of the formulation and introducing functional adhesive groups are critical factors for prolonging the retention time on the oral mucosa. These strategies help to increase the effective contact area with mucosal tissues and achieve synergistic interplay of multiple adhesion mechanisms, thereby meeting the requirements of ideal formulation design. Meanwhile, the introduction of stimuli-responsive materials provides new technological avenues for oral drug delivery system design. In situ forming formulations, such as thermosensitive and photocurable systems, can cover oral wounds in the form of powders or droplets. Compared with preformed patches or films prepared in vitro, they offer higher interfacial conformability and coverage efficiency, and are expected to promote wound healing. Furthermore, facile fabrication techniques, including vacuum drying and electrospinning, further augment the clinical translation potential of these formulations.

Notwithstanding the considerable promise exhibited by the aforementioned advanced formulations in preclinical investigations, their clinical translation remains encumbered by several potential immunogenic risks. It has been demonstrated that reactive dialdehyde polysaccharides elicit a disruption of fibroblast structural integrity, concomitant with a diminution of cellular metabolic activity [158]. In accordance with the hazard statements of the Globally Harmonized System of Classification and Labeling of Chemicals (GHS), N-hydroxysuccinimide (NHS) active esters, acrylic acid, and polyethyleneimine are explicitly classified as skin irritants (H315) and respiratory irritants (H335). Dopamine and levodopa, which serve as representative catechol-based adhesive moieties, have also been revealed to carry potential neurotoxic risks. Furthermore, the multiple irritant effects of ultraviolet (UV) light on oral mucosal cells have been extensively documented, including marked inhibition of cell proliferation, upregulation of cyclooxygenase-2 (COX-2) mRNA expression, and enhanced generation of prostaglandin E2 (PGE2), collectively contributing to the induction of local inflammatory responses in the oral cavity [159,160]. Although certain active materials, such as tannic acid and lipoic acid, exhibit favorable anti-inflammatory properties and low immunostimulatory potential, they have not yet received formal approval from the European Union or the United States Food and Drug Administration (FDA). Consequently, when employed in the development of oral mucosal formulations, a comprehensive assessment of their risk–benefit profile remains indispensable to ensure the maximization of therapeutic benefit.

### 4.2. Strategies for Enhancing Penetration

The mucus layer and epithelial layer will impede drug penetration. During crossing the barrier, the drug’s penetration rate significantly decreases and is trapped here. Therefore, it is essential to employ permeation enhancement strategies to improve absorption during drug formulation design. Currently, permeation enhancement strategies can be categorized into traditional and novel permeation enhancement methods.

#### 4.2.1. Traditional Permeation Enhancers

Traditional permeation enhancement methods often involve the addition of chemical substances such as surfactants, bile salts, fatty acids, cell-penetrating peptides, and cyclodextrins to the preparation. Surfactants and bile salts, serving as highly effective permeation enhancers, have been widely employed to improve the permeability of various drugs through the oral mucosa. These permeation enhancers facilitate drug penetration by forming micelles, chelating calcium ions, and dissolving mucus [161,162,163,164]. Table 5 summarizes the classification, usage concentrations, mechanisms of action, and toxicity of commonly used traditional permeation enhancers. Numerous studies have demonstrated that the use of permeation enhancers significantly enhances the permeability and absorption efficiency of drugs. Kurosaki et al. investigated the effects of sodium lauryl sulfate (SLS), cetylpyridinium chloride (CPC), polysorbate 80 (PS-80), and sodium taurocholate (STC) on the oral mucosal absorption of salicylic acid [108]. Results indicated that SLC and CPC accelerated salicylic acid absorption, whereas PS-80 inhibited its absorption. Oleic acid is a common fatty acid permeation enhancer that has been applied to enhance the oral mucosa permeation of lipophilic drugs, which disrupts the ordered arrangement of lipids and enhances drug permeation via nonpolar pathways [165,166]. The permeation-enhancing effect varies among fatty acids, primarily determined by different factors such as degree of unsaturation, chain length, ionization state, and isomer type [167]. Padula et al. reveal a parabolic relationship between fatty acid lipophilicity and permeation enhancement [165]. Morishita et al. demonstrated that oleic acid-containing insulin exhibits superior drug release kinetics and therapeutic efficacy [107]. Although the above compounds demonstrate excellent permeation-enhancing effects and low cost, safety concerns remain a significant issue [85]. The most pressing safety concerns involve irritation and damage to mucosal tissues, as well as the recovery process following damage. In particular, permeation-promoting agents such as surfactants and bile salts operate through mechanisms that involve disruption of intercellular tight junctions or reduction in mucus viscoelasticity. When used to treat oral mucosal disorders, the compromised epithelial barrier in affected areas increases the absorption of drugs and permeation enhancers; however, this can conversely raise the risk of toxicity and cause a sharp increase in drug concentration, ultimately reducing the therapeutic efficacy. Therefore, when these permeation enhancers are considered, dosage concentrations must be carefully controlled to ensure ideal permeation effects and safe administration. Meanwhile, recent findings suggest that cell-penetrating peptides (CPPs) may be novel mucosal permeabilizers attributed to biocompatibility, membrane permeability, low toxicity, and low immunogenicity [168,169,170]. Nevertheless, few studies have explored the permeation-enhancing effects of CPPs on oral mucosa. The field of CPP-mediated oral mucosal drug delivery remains relatively underdeveloped, yet holds immense application potential.

#### 4.2.2. Novel Permeation-Enhancing Methods

Given the inherent drawbacks of traditional permeation enhancers, scientists have progressively explored novel permeation enhancement methods to minimize toxicity and damage to mucosal tissues. Microneedles and nanomedicines represent the most extensively researched and applied novel permeation enhancement approaches.

##### Microneedle

Microneedles represent a novel minimally invasive device. They consist of two components: an adhesive patch and a micron-scale needle array [167]. Painless and convenient characteristics have rapidly gained patient favor. As an innovative drug delivery system, microneedles demonstrate distinct advantages in oral mucosal drug delivery. Precise administration and minimal invasiveness are two primary advantages of microneedle drug delivery. Conventional patches, gels, and powders suffer from slow drug permeation, requiring extended retention. In contrast, microneedles can break through the penetration barrier and deliver the drug directly to the deep tissues. As early as 2015, Ma et al. prepared doxorubicin (DOX)-loaded coated microneedles for oral cancer treatment [178]. When the microneedle patch was applied to the oral lesion, DOX was constantly permeated and uniformly distributed, obviously enhancing tissue absorption and bioavailability. Since then, microneedle technology has gradually emerged as an alternative approach for oral drug delivery and oral disease treatment, such as oral ulcers, oral cancer, and oral mucositis. However, given the unique physiological structure of the oral mucosa, the design of microneedles is particularly crucial. Here, we list some important factors that need to be considered when designing microneedles.

(1) When puncturing the oral mucosa, the robust mechanical property is a critical factor in ensuring the structural stability of the microneedle and a successful puncture. Softer microneedles may fail to fully penetrate the barrier layer, resulting in incomplete drug delivery. For instance, Xiao et al. incorporated nanoenzymes into microneedles (DC@MN), significantly enhancing the mechanical strength (Figure 7a) [109]. HE staining revealed that DC@MN successfully penetrated the epithelium, whereas PVP-HAMA-MN, which did not contain nanozymes, failed to do so (Figure 7b). Furthermore, DC@MN could withstand higher forces without rupture at the same displacement (Figure 7c). (2) An appropriate microneedle length is critical for achieving efficient therapeutic outcomes. When the microneedles are too short, only a negligible amount of the released drug reaches the target site. Conversely, excessively long tips may aggravate the disease condition. Santos et al. indicate a positive correlation between microneedle length and pain intensity, and the pain sensitivity differs at various insertion sites [179]. It should be emphasized that the microneedle length should be matched to the specific anatomical site of the lesion when designing microneedles. This consideration is based on the structural differences and pathological variations across different regions of the oral mucosa. Although this factor is rarely taken into account in conventional permeation enhancement strategies, the mechanism of microneedle-mediated enhancement primarily involves physical disruption of the epithelial layer, and therefore mucosal variability must be taken into consideration. Under normal physiological conditions, the buccal mucosa possesses the thickest epithelial layer, as it is involved in mastication; a thicker epithelium helps buffer masticatory forces and reduce chemical irritation. In contrast, the sublingual and floor-of-the-mouth mucosa are the thinnest, with rich vascularity and high permeability, and excessively long tips may even pose a risk of bleeding. These differences in oral mucosal thickness are consistent with findings reported in multiple studies [180,181,182]. Under different pathological conditions, the thickness of the oral mucosa also varies due to differences in disease pathogenesis. Several studies have indicated that diabetes-associated oral ulcers and oral mucositis tend to cause a decrease in oral epithelial thickness, whereas oral leukoplakia and oral squamous cell carcinoma are more likely to induce keratinization and mucosal thickening [183,184,185,186]. Therefore, microneedle length design must be adapted to the thickness of the oral epithelial layer in order to achieve effective therapy and successful penetration. Zhang et al. developed an acetylpolysaccharide-loaded soluble microneedle patch (APS-MN) for treating the oral ulcers in buccal mucosa [187]. HE staining revealed microneedle puncture depths up to 400 μm (Figure 7d). After six days of treatment, the APS-MN group achieved an 88.2% wound healing rate, significantly higher than other groups (Figure 7e). Ma et al. prepared curcumin analog-loaded microneedles for OSCC treatment [188]. The fabricated microneedle tips are up to 590 μm in length, ensuring successful penetration and subsequent drug release. (3) The tip geometry ensures drug localization at the lesion site. For immediate-release microneedles, their rapidly dissolving nature enables prompt drug release. However, for sustained-release microneedles, the elastic force of the mucosa may exert an upward force on the microneedles, making it difficult to achieve sufficient insertion depth. Therefore, some researchers have adopted tip swelling and barbed anchoring strategies to ensure microneedle fixation. Liu et al. have detailed these two fixation strategies, which can guarantee adequate retention, enabling controlled drug release and intervention in the disease process [189].

When the oral mucosa is punctured, drugs will escape from the microneedles and diffuse into tissues, which is also regarded as drug permeation. Adjusting the material composition may be an option to regulate the drug release curve to achieve ideal permeation and therapeutic effects. Based on this assumption, numerous intelligent microneedles have been successfully engineered. Generally, the needle tip serves as the primary region for drug loading. The needle can be vertically divided into the tip and base layers, or horizontally categorized into the core and shell layers. Drugs at distinct layers exhibit different release rates. The tip and shell layers dissolve more rapidly due to earlier contact with tissue fluid. For instance, Lee et al. take the ceria nanoparticles (mEI-Ce) and triamcinolone acetonide (TA) into the tip and base layer, respectively, realizing comprehensive treatment for oral ulcers [190]. In vitro drug release profiles detected a burst release of mEI-Ce within the first 30 min, while TA showed sustained release over 20 h. When microneedles were applied to mucosa, most fluorescence was observed in ulcerative tissue, indicating the released drug had permeated and been absorbed by the pathological site. Tang et al. developed core–shell microneedles loaded with lidocaine and dexamethasone for treating oral ulcers (Dex@MNs) [191]. Upon insertion into the oral mucosa, 72% of the shell lidocaine was released within 1 min, while the core dexamethasone was released in a sustained manner for 24 h (Figure 7f,g). After 6 days of treatment, ulcer areas in the Dex@MNs group seem to have disappeared (Figure 7h). Material selection also influences drug release. Non-crosslinked and simple materials exhibit excellent dissolvability. Conversely, crosslinked and composite materials resist dissolution and drug release due to enhanced molecular interactions. For instance, Meng et al. engineered a core–shell microneedle patch loaded with lidocaine and betamethasone for oral ulcer treatment [110]. Lidocaine was encapsulated within the internal HA layer, which is rapidly dissolved by tissue fluid, ensuring instant analgesia. The betamethasone encapsulated in the HAMA layer (outer layer) was released slowly, exerting a potent anti-inflammatory effect. At the diffusion time of 6 h, the amount of lidocaine released reached 90%. The amount of BT released in the slow-release drug model was approximately 60%. With the growing popularity of photothermal therapy, responsive microneedles combined with phototherapy can significantly enhance drug release and diffusion. Song et al. indicate that microneedles exposed to infrared light exhibit greater fluorescence diffusion [192]. Furthermore, the data show that the length, width, and average intensity of the fluorescent zone are significantly enhanced after near-infrared irradiation (Figure 7i). The iontophoresis technique provides transmucosal administration of drugs by electromigration and electroosmosis. Research demonstrated that the dye-covered area in the MN patch–iontophoresis group was twice that of the control MN group [193].

Microneedles represent a novel pharmaceutical delivery system whose minimally invasive and precise characteristics attract patient attention. An increasing number of researchers are focusing on achieving oral mucosal drug delivery through microneedles to overcome the permeability barrier. When microneedles are designed, their structure is the primary factor considered, as it determines the penetration depth and fixation time. Secondly, drug distribution and microneedle materials also contribute to desirable release and penetration effects. The application of combined therapies has significantly enhanced drug penetration. Therefore, when the permeability of microneedles is expected to increase, multi-dimensional analysis is required, which encompasses material composition, structural design, and combined therapies.

**Figure 7:**
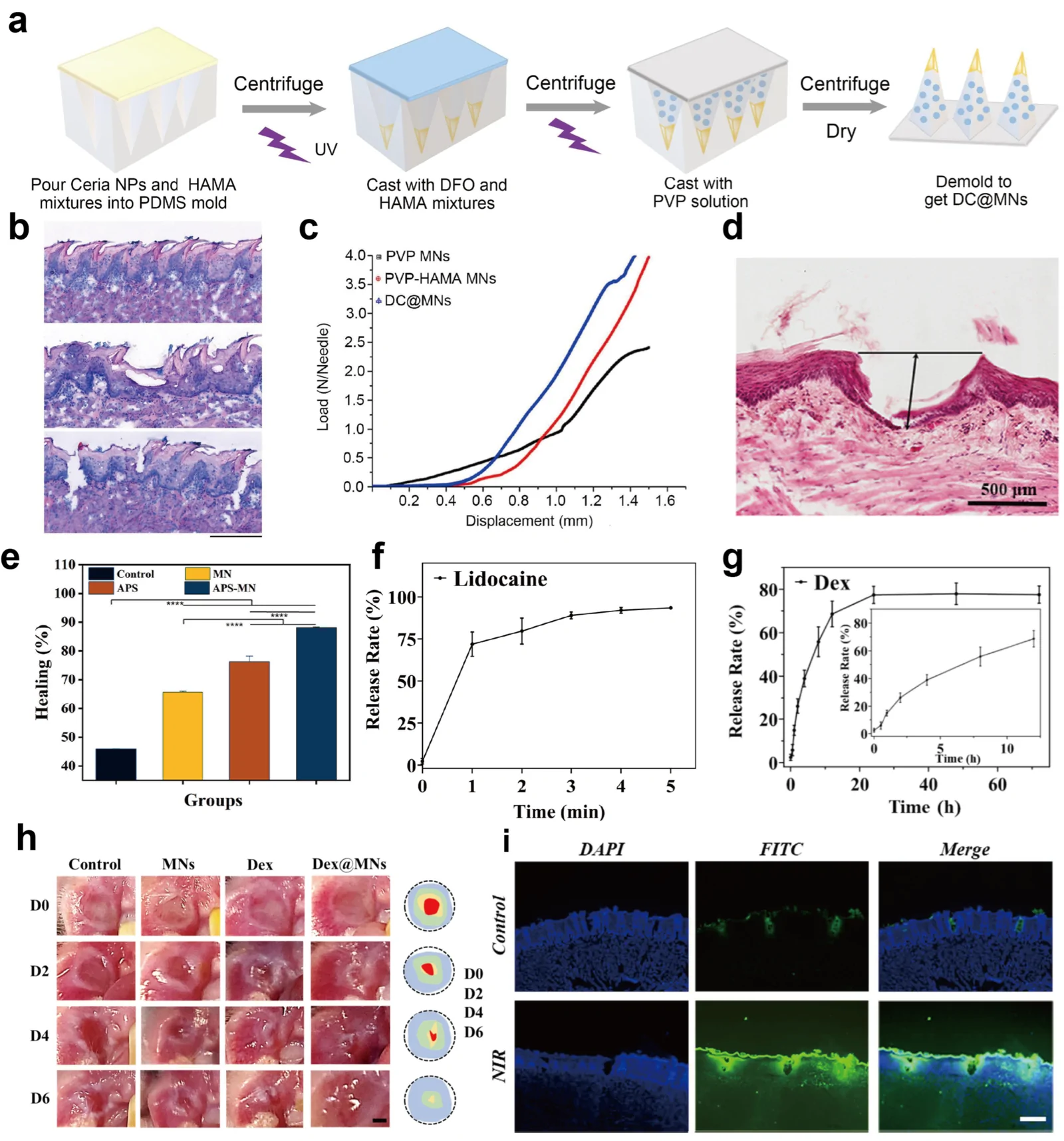
Microneedle drug delivery system for application to the oral mucosa. (**a**) Schematic diagram of the DC@MNs preparation process. (**b**) The H&E-stained images show the epithelial structure of the tongue dorsum in a normal rat (**top** image), the structure of the tongue dorsum following the administration of PVP-HAMA MNs (**middle** image) and DC@MNs (**bottom** image). The scale bar is 250 μm. (**c**) Compression–displacement curves of the PVP MNs, PVP-HAMA MNs, and DC@MNs groups. (**d**) HE-stained image showing APS-MN patches after puncture of the oral mucosa. (**e**) Comparative analysis of wound healing rates between groups after 6 days of treatment. *p* values < 0.05 were considered to be significant (**** *p* < 0.0001). In vitro release profiles of lidocaine (**f**) and dexamethasone (**g**) from MNs. (**h**) The left section displays images of wound healing in different treatment groups at specific time points. Scale bar = 1 mm. The right section shows the corresponding results of wound area tracking analysis. (**i**) Fluorescence images showing dye diffusion in tissue with and without NIR application. Scale bar = 100 μm. Panels (**a**–**c**), Panels (**d**,**e**), Panels (**f**–**h**) and Panel (**i**) adapted with permission from Refs. [109,187,191,192]. Copyright © Adapted from Elsevier and Wiley.

##### Nanoparticle Delivery System

The rapid evolution of nanotechnology has significantly accelerated progress in medicine. Micro- and nano-delivery systems have a place in drug delivery. For the treatment of various solid tumors, nanoparticle delivery systems have gradually taken over chemotherapy and radiotherapy due to the enhanced permeability and retention (EPR) effect. In our previous work, we discussed the advantages of nanomedicine delivery systems for tumor therapy [194]. Although nanomedicine research is primarily concentrated in tumor treatment, our investigation reveals that nanoparticle systems have already been applied to mucosal penetration. Early in 2009, Hearnden et al. conducted oral mucosal diffusion studies of nanometer polymersomes assembled from phosphorylcholine, ethyl methacrylate, and poly(ethylene oxide) [195]. Subsequent research revealed that the surface properties of nanoparticle systems have a significant impact on mucosal penetration. Subsequent studies have revealed that the surface properties of nanoparticle systems significantly affect mucosal permeability. This review summarizes the key characteristics that influence the permeability of nanoparticle systems, including particle size, shape, surface properties, and mucus properties.

(1) Particle size: The penetration of nanoparticles across the oral mucosa does not follow a simple linear relationship with particle size; rather, it exhibits an optimal size range within which the highest penetration efficiency is achieved. The gel network covering the mucosal surface constitutes a physical barrier with size-sieving properties. From the perspective of penetration kinetics, when the nanoparticle size is substantially smaller than the average mesh size of the network, the particles are geometrically capable of traversing the pores. However, this theoretical prediction is often not borne out in practice. Lai et al. observed that 100nm particles migrated significantly slower than 200nm and 500nm particles [196]. This finding is not coincidental. In vitro permeation studies conducted on porcine buccal mucosa by Teubl et al. revealed a conclusion that is contrary to the anticipated size-dependent trend. The 200 nm neutral polystyrene particles, among the three sizes tested (25, 50, and 200 nm), displayed the highest penetration velocity and the deepest tissue penetration [197]. Owing to larger specific surface area and higher surface energy, smaller nanoparticles are prone to nonspecific interactions with mucins, leading to immobilization and hindering diffusion to the epithelium. In contrast, particles exceeding the mesh size are physically entrapped by the fibrous network and cannot reach the epithelial layer. Although no optimal size range has been established, 100–300 nm is generally considered favorable for oral mucosal penetration [197,198,199,200]. (2) Particle shape: Size selection is crucial in nanoparticle design. Elasticity and shape also need to be considered. Different shapes exhibit distinct hydrodynamic behaviors. Clubbed and linear particles exhibit looser structures and greater flexibility. These characteristics enable them to alter spatial conformation and readily deform to pass through a pore [201,202]. In contrast, when spherical particles collapse upon themselves, the intermolecular interactions and steric hindrance limit their deformability. Thus, spherical particles will encounter greater resistance when passed through a pore. As Bao and colleagues in research noted, nanotubes can penetrate faster and deeper than nanospheres in mucus [203]. Yu and his colleagues also revealed that the superior mucus penetration capability of nanotubes stems from rotational motions of the nanorods [204]. The inherent flexibility and rotational motion of nanotubes enable faster penetration through the mucus layer to reach the target site. (3) Particle surface properties: Surface characteristics govern mucosal penetration. Inert coatings (e.g., PEG) reduce mucin adhesion and promote rapid transit. Yamazoe et al. found that PEGylated liposomes showed concentration-dependent mucosal permeability, while glycol chitosan (GCS)-modified ones were impeded by mucus [205]. Surface charge is also commonly considered a cue for mucin entrapment. Sialic acid, a major component in mucins, exhibits strong affinity for cationic nanoparticles, trapping them within the mucus layer. Conversely, anionic or neutral particles successfully evade sialic acid capture and penetrate the mucus layer [206]. However, cellular binding affinity for anionic nanoparticles is significantly weaker than for cationic ones. Charge-reversible nanoparticles have been designed to overcome this barrier: after mucus penetration, they switch charge to promote endocytosis. Wu et al. co-modified surfaces with the R8 peptide and phosphoserine. The negative charge facilitated mucus transit, while enzyme-triggered reversal at the epithelium exposed cationic R8 to drive cellular uptake and transepithelial transport [207]. (4) Mucosal status: Mucosal condition critically affects nanoparticle penetration. Pathological states vary. Diseases such as oral submucous fibrosis and candidiasis cause epithelial thinning and enhanced penetration, whereas oral lichen planus increases saliva viscosity and mucus density, impeding penetration. Hence, formulation design must account for disease-specific mucosal alterations to optimize therapeutic efficacy.

Numerous examples of nanoparticle systems have also been applied in oral formulations. Particularly to reduce the use of traditional permeation enhancers, many researchers adopt nanoparticle systems to enhance drug penetration. For instance, Hu et al. evaluated the permeation of dexamethasone-loaded nanoparticles, composed of PLGA, PEG, PDA, and PVA, through oral mucosa [155] (Figure 8a). Compared to other nanoparticles, PLGA-PDA exhibited an 80% penetration rate within 3 h (Figure 8b). Figure 8c shows that the displacement of PLGA-PEG and PLGA-PDA increased over time, indicating their diffusion and movement within the mucus layer. In contrast, the displacement of PLGA-PVA and PLGA is relatively static, suggesting they may have been trapped within the mucus layer. To clarify this situation, the authors mixed nanoparticles and mucin suspension to evaluate binding capacity. As shown in Figure 8d,e, PLGA and PLGA-PVA nanoparticles exhibited gradual increases in particle size and decreases in zeta potential over time, indicating interaction with mucin. In contrast, PLGA-PDA and PLGA-PEG maintained relatively stable particle size and zeta potential, suggesting weaker binding affinity. Matos et al. encapsulated oxaliplatin (OXPt) in chitosan nanoparticles for topical oral tumor treatment [111]. In vitro permeability experiments demonstrated that nanoparticles significantly enhanced drug penetration. After 2 h, drug absorption increased approximately threefold (Figure 8f). Friedl et al. developed a self-emulsifying drug delivery system (SEDDS) for oral administration of the poorly soluble drug curcumin (Figure 8g) [208]. The engineered curcumin-SEDDS exhibited a size of 29.1 nm. Tissue section analysis evaluated curcumin penetration, where SEDDS significantly increased curcumin permeability (Figure 8h). Curcumin-SEDDS showed not only an extensive penetration into the epithelium but also into deeper layers of the mucosa. Xiang et al. utilized the enhanced penetration principle to incorporate melanin nanoparticles into a polysaccharide hydrogel (GCM3), combined with photothermal therapy for bacteria-infected diabetic wound healing management [209]. After 5 days of treatment, the ulcer area in the GCM3+NIR group had healed almost completely (Figure 8i). Zhou et al. adopted DNA tetrahedrons (TDN) as molecular carriers for miR132 delivery (Figure 8j) [210]. The unique triangular pyramidal structure exhibits excellent tissue penetration and cellular uptake, facilitating efficient drug penetration and release. After 7 days of treatment, the wound area of oral mucosa was reduced to 29.86%, significantly lower than that of commercial oral patches (65.97%) and the control group (69.57%) (Figure 8k).

Nanoparticle systems offer novel approaches for mucosal barrier penetration. Owing to inherent properties, nanomaterials for mucosal permeation applications are increasingly prevalent. Nanoparticle systems overcome many challenges that traditional drug delivery systems faced for mucosal administration, such as retention, permeation, and endocytosis. Appropriate parameters are crucial for the release and penetration of the drug and nanocarrier, particularly within the complex, dynamic, and moist oral cavity.

**Figure 8:**
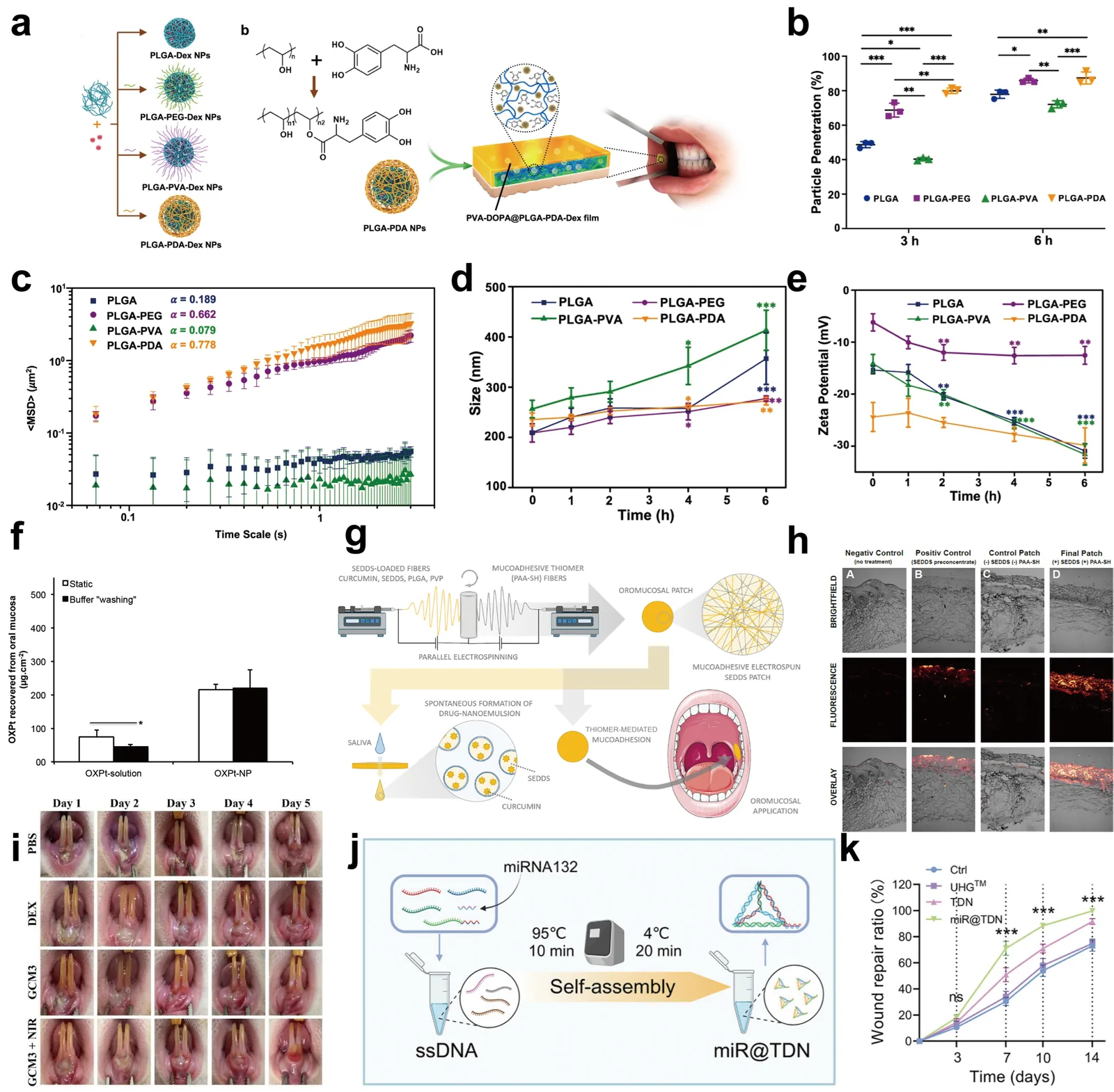
Nanoparticle drug delivery system for oral mucosal application. (**a**) Schematic diagram of core–shell PLGA nanoparticles with different surface modifications and the preparation of PVA-DOPA@NPs-Dex films. (**b**) Percentage of NPs that penetrated across the mucus layer in a Transwell assay after 3 h and 6 h. (**c**) MSD (mean squared displacement) values as a function of time scale for different NPs in mucus. Variation in particle size: (**d**) zeta potential and (**e**) different nanoparticle-mucin mixtures as a function of time. *p* values < 0.05 were considered to be significant (* *p* < 0.05, ** *p* < 0.01, *** *p* < 0.001). Panels (**a**–**e**) adapted with permission from Ref. [155]. Copyright © Adapted from Springer Nature. (**f**) The 2 h of treatment with OXPt-NP in comparison to control (OXPt aqueous solution) followed by the static and buffer “washing” protocols. (**g**) Schematic diagram of the preparation process for curcumin-loaded mucoadhesive electrospun SEDDS patch and its application to the oral mucosa. (**h**) Bright-field, fluorescence, and overlay images of buccal mucosa cross-sections without treatment (**A**) or after incubation with curcumin formulated either in the form of emulsified SEDDS preconcentrate (**B**) and electrospun fiber patches without both SEDDS preconcentrate and thiolated PAA (**C**), or the final fibers comprising parallel electrospun SEDDS fibers and mucoadhesive PAA-SH fibers (**D**). (**i**) Photographs of ulcer healing on days 1–5 after treatment in the PBS, 3M, GCM3, and GCM3 + NIR groups. Panel (**f**), Panels (**g**,**h**), and Panel (**i**) adapted with permission from Refs. [111,208,209]. Copyright © Adapted from Elsevier. (**j**) The self-assembly process of miR@TDN. (**k**) Percentage of wound healing area in different treatment groups at various time points. *p* values < 0.05 were considered to be significant (*** *p* < 0.001, ns, not significant). Panels (**j**,**k**) adapted with permission from Ref. [210]. Copyright © Adapted from John Wiley and Sons.

### 4.3. Strategies for Enhancing Adaptability to Mechanical Environments

Adhesion and permeation enhancement strategies provide preliminary solutions for drug delivery. However, the oral mucosa resides in a continuously dynamic environment, where activities such as chewing, swallowing, and speaking impose multiple mechanical stresses on pharmaceutical formulations. An ideal drug delivery system for the oral mucosa must possess appropriate mechanical strength to resist structural disruption, exhibit self-healing properties to extend its service life, and avoid adhesion to non-target tissues to ensure therapeutic efficacy.

#### 4.3.1. Improving Mechanical Properties

The core objective of enhancing mechanical properties is to improve the polymer network’s resistance to deformation and disruption, primarily achieved through the incorporation of high-modulus materials and increased crosslinking density. Firstly, the uniform dispersion of functional nanomaterials within the polymer matrix can significantly enhance the overall mechanical performance of the composite material. This enhancement is attributed to the nanomaterials’ high stiffness, large specific surface area, and interfacial interactions with the polymer matrix, which facilitate load transfer. Nanomaterials currently employed in this field primarily include metal nanoparticles, natural polyphenol nanoparticles such as tannic acid, ellagic acid, curcumin, nanocellulose, and MXene two-dimensional nanomaterials. Yi et al. developed a multifunctional hydrogel patch based on gold nanostars [112]. By introducing gold nanostars into a polyacrylamide (PAAm) hydrogel network, they leveraged their high stiffness for mechanical reinforcement, resulting in an increase in tensile strength at break from approximately 14.356 MPa in the pristine state to 18.280 MPa, and a more than threefold increase in tensile fracture strain (Figure 9a,b). Fang et al. designed a novel hydrogel patch incorporating tannic acid particles [113]. They compared the properties of hydrogels containing different particle concentrations, namely POQ0, POQ1, and POQ2 at concentrations of 0, 0.5, and 1 mg/mL, respectively. Results showed that compared to POQ0, which had an average pore size of 49.2 ± 4.8 μm, the average pore size of POQ2 decreased to 25.2 ± 9.1 μm, and its rupture stress reached a higher 38.6 kPa compared to 28.7 kPa for POQ0 (Figure 9c,d). Combined with scanning electron microscopy (SEM) results, this indicated that increasing tannic acid concentration led to a denser porous structure within the hydrogel and more uniform embedding of poly(tannic acid) (PTA) particles. This enhanced interfacial interactions such as hydrogen bonding, hydrophobic interactions, and π-π stacking between tannic acid and polymer chains, thereby improving the hydrogel’s resistance to deformation. Secondly, crosslinking density is a core parameter determining the mechanical properties of polymers. Whether through ionic, covalent, or physical crosslinking, increasing crosslinking density effectively counteracts mechanical stress. To address the issues of commercial mucoadhesive films being prone to rupture and exhibiting low drug utilization in the dynamic, moist oral environment, Chang et al. designed a mucoadhesive film based on Ca^2+^-crosslinked carboxymethylated cellulose nanofibers and alginate [114]. By utilizing the coordination interaction between Ca^2+^ and carboxyl groups on CMCNFs and SA, they systematically modulated the ratios of Ca^2+^, sodium alginate, and carboxymethyl cellulose to increase the number of crosslinking points within the polymer network, constructing a high-density ionic crosslinking network. Data revealed that this wet-state film exhibited a Young’s modulus of 7.1 ± 2.6 MPa and a strain at break of 53.6 ± 9.8%, enabling resistance to deformation caused by frequent oral movements while demonstrating excellent water durability and biocompatibility. This design achieved a balance between mechanical strength and flexibility, avoiding both tissue damage from excessive rigidity and structural disruption from excessive softness. Wang et al. constructed an interpenetrating polymer network (IPN) by combining the covalent crosslinking network of the GelMA network, serving as a rigid skeleton, with the physical entanglement network formed by polymer chains in M-HA [115]. Tensile testing revealed that the GelMA hydrogel had a relatively low elongation at break of approximately 40%, whereas the GelMA/M-HA hydrogel showed a significantly increased elongation at break of approximately 70%, indicating superior toughness and extensibility suitable for the dynamic movement requirements of the oral mucosa (Figure 9e). SEM results showed that the GelMA/M-HA hydrogel exhibited a denser and more uniformly porous network structure compared to pure GelMA hydrogel (Figure 9f), providing a favorable structural basis for its application in the mechanically dynamic oral environment.

#### 4.3.2. Enhancing Self-Healing Properties

Self-healing refers to a material’s ability to autonomously or under external stimulus restore its structure and function after damage, primarily achieved through dynamic chemical bonds or reversible physical interactions. Dynamic covalent bonds, such as Schiff bases, acylhydrazone bonds, disulfide bonds, and borate esters, combine the stability of covalent bonds with the dynamism of reversible bonds, representing a classic strategy for achieving self-healing. Su et al. rapidly constructed a novel hydrogel adhesive (CFT hydrogel) using a one-step method based on reversible Schiff base and borate ester bonds forming dual dynamic crosslinks [116]. The successful formation of these dynamic covalent bonds was confirmed through techniques such as UV–Vis spectroscopy. Cyclic strain sweep tests demonstrated that the storage modulus (G′) could instantly recover to near its initial value upon switching from high to low strain, remaining consistently higher than the loss modulus (G″), with the two lines maintaining near-parallelism in each cycle (Figure 9g). This rapid modulus recovery evidenced the excellent reversible reorganization capability of its network structure. Notably, the G′/G″ value of the C5F1.4T1.6 group was significantly higher than those of the T0 and T0.8 groups, confirming that the addition of TA (Tannin) facilitated the formation of more dynamic covalent bonds within the hydrogel, enhancing the reversible transition ability of the crosslinked network from disruption to reconstruction. This design enables the hydrogel to spontaneously repair its network structure after damage, thereby maintaining its integrity and function within dynamic environments like the oral cavity. Sun et al. designed an Fe-TA complex-modified P(AM-AA) hydrogel (Fe-TA@P(AM-AA)) [117], utilizing the dynamic complexation-dissociation process of the Fe-TA complex to provide impetus for the repair of the gel network, assisting the gel in restoring structural integrity after damage. Strain amplitude sweep and cyclic strain sweep experiments showed that the collapse and reorganization process of the gel network could be repeated multiple times under high strain of 1000% and low strain of 10% (Figure 9h), indicating the excellent self-healing ability of the prepared hydrogel. Cut-and-heal tests confirmed that the fractured hydrogel could restore its initial overall morphology in just 5 min, and the healed hydrogel could be stretched, further demonstrating its outstanding self-healing performance (Figure 9i).

#### 4.3.3. Avoiding Adhesion to Non-Target Tissues

However, while excellent mechanical strength and self-healing capabilities ensure the structural stability of formulations within the dynamic oral environment, this only addresses the mechanical adaptability of the bulk material. In practical clinical application, formulations must adhere firmly to ulcer wounds to exert therapeutic effects, while simultaneously avoiding unintended adhesion to non-target tissues such as the tongue and buccal mucosa. The latter not only induces significant foreign body sensation and discomfort but may also lead to premature detachment, thereby undermining the clinical benefits conferred by the previously optimized mechanical properties. Asymmetric surface design simultaneously balances lubrication and adhesion functions, reducing food attachment and tongue adhesion while promoting drug retention. Cheng et al. integrated two completely opposing interfacial properties into a single patch through an asymmetric bilayer design, combining a strong wet-adhesive layer based on silk fibroin-tannic acid (SF-TA) with a non-adhesive treatment layer based on pure silk fibroin (SF) [120]. In comparative adhesion tests, the adhesion strength between the SF-TA layer and a non-adhesive SF handling film was as high as 94.54 kPa (Figure 9j), while the adhesion force between the SF layer and the SF handling film was only 0.38 kPa, a difference of approximately 250-fold, which means that this asymmetric design effectively prevents accidental adhesion between oral mucosal preparations and non-target tissues such as the tongue. Inspired by the multilayered asymmetric structure of natural mucosa, Zhang et al. designed a Janus hydrogel patch designated ANSB [118]. The zwitterionic nature of its lubricating layer provides excellent lubrication and inherent antifouling properties, achieving a low coefficient of friction of 0.02 ± 0.003 (Figure 9k). This closely mimics the lubricating characteristics of natural mucosal tissue, effectively preventing bacterial invasion, secondary injury, and unintended adhesion to non-target tissues. Distinct from the Janus bilayer structure, Zhang et al. developed a strong wet-adhesive hydrogel based on mussel-inspired chemistry [119], which reduces unintended adhesion to non-target tissues through mechanisms of surface hydrophobization and selective adhesion. In this formulation, negatively charged catechol-functionalized hyaluronic acid and positively charged polyethyleneimine form a polyelectrolyte network, triggering a chain rearrangement and directional surface aggregation of cholesterol-containing micelles, thereby constructing a hydrophobic hydrogel surface (Figure 9l). Data indicated that the control hydrogel exhibited a water contact angle of approximately 20° (Figure 9m), indicating hydrophilicity, whereas the surface engineered via cholesterol micelle rearrangement showed a significantly increased water contact angle of 80–90°, confirming the successful construction of a hydrophobic surface layer. This layer itself exhibits a certain degree of repellency towards water molecules and hydrophilic biomacromolecules such as mucins in saliva, displaying low affinity upon contact with healthy, moist non-target tissues. It reduces the non-specific adsorption of salivary mucins, cellular debris, and microorganisms onto the material surface, thereby mitigating the risk of secondary adhesion mediated by contaminants at its source and improving drug bioavailability.

## 5. Conclusions and Perspectives

The oral mucosa is a soft tissue within the oral cavity that plays a critical role in oral health and function. Inflammation, infection, and tumors can lead to mucosal lesions, resulting in physical and mental suffering for patients. Numerous oromucosal products are available on the market. However, their therapeutic efficacy remains unsatisfactory. These formulations appear to face similar challenges, including poor adhesion, inadequate penetration depth, and fragile formulation properties. To address these issues, scientists have developed several strategies targeting adhesion, penetration, and formulation enhancement to improve drug retention and diffusion. Adhesion strategies aim to reduce interfacial saliva and strengthen interfacial interactions, thereby extending drug retention at the mucosa. Penetration strategies involve modulating epithelial permeability and structure to enhance drug deposition. Formulation optimization strategies address the challenges posed by complex mechanical stresses in the oral cavity. Enhanced mechanical properties, self-healing capabilities, and asymmetric structure offer new possibilities. These strategies provide a new paradigm for developing next-generation oromucosal formulations.

Nevertheless, the development of oromucosal formulations still encounters inevitable obstacles arising from potential risks inherent in new strategies and from challenges existing during the transition from the laboratory to industrial and clinical application. In this section, we systematically analyze these specific challenges. (1) The biosafety of next-generation oromucosal formulations is a serious concern. Despite extensive academic efforts to develop novel strategies that overcome the limitations of commercial products, regulatory approval and clinical translation remain exceedingly rare. Biosafety and regulatory clearance have thus emerged as primary developmental hurdles. As noted earlier, polymer-induced immunostimulation, functional group-associated immunological risks, and residual manufacturing toxicants significantly hinder the approval of next-generation formulations. Nevertheless, these risks represent only part of the challenge. A more profound predicament is the systemic absence of methodologies to assess long-term and comprehensive biomaterial safety profiles. Although ISO 10993 [211] provides a biocompatibility foundation, its test methods inadequately predict true in vivo responses, often overlooking clinically relevant endpoints such as infection, foreign body response, and fibrosis. Moreover, prevailing in vitro systems rely on two-dimensional cell models and short-term observations (typically 24–72 h), failing to capture extended host–material interactions. For oral mucosal formulations requiring prolonged retention and repeated administration, current paradigms cannot adequately evaluate local immune dysregulation and systemic exposure risks manifesting over weeks to months. These methodological shortcomings constitute a structural evaluation bottleneck, depriving regulators of sufficient scientific evidence for rigorous product review and critically impeding the translational advancement of next-generation formulations from bench to bedside. (2) The drug release cycle does not correspond with the entire disease course. On the one hand, the reported drug retention time rarely exceeds 48 h, which is insufficient to cover the entire disease course. On the other hand, despite current research often integrating multiple drugs into the formulation, there is a lack of studies on phased drug release. For example, the course of acute oral ulcers is approximately 7–14 days, involving inflammation, healing, and remodeling. Although novel oromucosal formulations integrate anti-inflammatory and pro-healing agents, they lack investigation into release sequences and timing. This unrefined drug delivery strategy struggles to achieve phased drug release, particularly during the healing and remodeling phases, where drug retention is virtually nonexistent. (3) The industrialization of next-generation oral mucosal drug delivery systems faces substantial hurdles, primarily stemming from uncertainty in regulatory classification. Within the current FDA framework, novel formulations may fall into four distinct categories, including drugs, medical devices, biological products, and combination products. These categories are governed by markedly different approval requirements. A New Drug Application (NDA) under Section 505 of the FD&C Act and 21 CFR Part 314 necessitates complete Phase I-III clinical trial data and comprehensive toxicological evaluations. Devices cleared via the 510(k) pathway (21 CFR Part 807) require demonstration of substantial equivalence, typically supported solely by bench performance testing, ISO 10993 biocompatibility assessment, and sterilization validation. A Biologics License Application (BLA) pursuant to Section 351(a) of the PHS Act and 21 CFR Parts 600/601 mandates cell line characterization, viral clearance verification, large-scale process validation, and immunogenicity monitoring. Combination products, governed by 21 CFR Part 3, are assigned to the lead review center based on their Primary Mode of Action (PMOA), with evaluation centered on integrated data packages for each component plus interaction studies. In practice, however, emerging formulations frequently resist unambiguous assignment to any single pathway. Chitosan, for example, Spossesses inherent mucoadhesive, biodegradable, penetration-enhancing, and antimicrobial properties, thereby conferring therapeutic efficacy even in the absence of co-delivered drugs. When such materials serve both as delivery matrices and pharmacologically active agents, classification becomes equivocal among drug, device, and combination product paradigms. Indeed, the FDA product classification database lists numerous chitosan-based wound dressings as “Unclassified”, reflecting a broader regulatory gap concerning devices composed of bioactive materials. Tannic acid and α-lipoic acid face analogous challenges. Although the FDA has codified PMOA as the determinative criterion for combination product jurisdiction, for chitosan-based oral mucosal formulations the therapeutic outcome may simultaneously arise from the pharmacological activity of loaded actives, the intrinsic anti-inflammatory and antimicrobial actions of the material itself, and its physical barrier function. This interplay precludes straightforward delineation of primary versus secondary mechanisms. Furthermore, the full-spectrum regulatory oversight of such products, including GMP compliance, supply chain traceability, and post-market surveillance, demands further refinement beyond the current regulatory infrastructure. (4) The requirement for personalized therapeutic strategies is growing. Disease progression varies significantly across different patient populations. The same formulation cannot be generalized to all groups. (5) Traditional preclinical models fail to accurately reflect human disease progression. Candidate formulations showing efficacy in rodent models often demonstrate suboptimal therapeutic outcomes during clinical application due to interspecies disparities in drug metabolism and the dynamic oral microenvironment.

In the above content, the application prospects and clinical translation of next-generation oromucosal formulations were analyzed and discussed. For existing challenges, future development directions and potential for next-generation oromucosal formulations were proposed. (1) Speeding up safety evaluation and regulatory approval for next-generation orrmucosal formulations. In the future, researchers should concentrate on safety research, which is an essential step toward clinical application. Given the unique oral microenvironment, a comprehensive biosafety evaluation system should be set up ranging from the safety of individual cells to tissues and even entire organisms. In addition, the progress of clinical trials and medication review of oromucosal formulations relies on the cooperation among legislators and regulatory bodies, scientific research institutions, and hospitals. (2) Machine learning (ML) will drive formulation optimization, which will come into trend in the future. With the development of AI technology, ML and deep learning are popular in the pharmaceutical field. To successfully deploy machine learning in oral mucosal formulations, it is imperative to comprehensively collect a broad spectrum of potential influencing parameters and incorporate them into the dataset, including solid content, concentration gradients, solvent categories, reaction conditions, instrumental parameters, in vitro pharmacodynamic data, in vivo pharmacodynamic data, and toxicological data [212,213]. For certain stringent preparation processes, environmental factors such as ambient humidity, temperature, and atmospheric pressure must also be incorporated into the dataset for model fitting, so as to prevent substantial deviations between the fitted formulation and the actual prepared product. External validation of the model should be performed using independent datasets derived from multiple different laboratories or confirmed through prospective experimental studies. In practice, machine learning can predict critical formulation properties, including mechanical strength, mucoadhesive strength, drug release kinetics, mucosal permeability, and formulation stability, thereby facilitating high-throughput screening prior to experimental execution. (3) The development of smart drug–biosensor integrated devices represents a new direction. These integrated devices act as intelligent microfactories, ensuring personalized and dynamic drug delivery through real-time monitoring, intelligent decision-making, and precise release. The transition of smart integrated devices demands not only sensor accuracy but also manufacturing reproducibility, biocompatibility of electronic components, and real-time signal stability within the oral environment. Clinical validation should be conducted in a stepwise manner, progressing from ex vivo porcine mucosa, animal models, or organoids to ultimately human trials, thereby establishing clear benchmarks for sensor sensitivity, drug release precision, and safety endpoints. (4) Alternative preclinical models will emerge as viable candidates to replace animal models. Recently, different types of contemporary 3D culture systems have been developed, including cell aggregates, organoids, and organ-on-a-chip devices. These system enabled through the integration of microfluidics, multi-fluid interfaces, multi-chamber design, and in situ monitoring, achieving better model environmental stimuli, tissue disease states, therapeutic efficacy, and systemic toxicity [214]. Novel preclinical models that more accurately recapitulate human disease states can bridge the physiological discrepancies between animals and humans with respect to the oral mucosa, thereby enabling direct evaluation of nanoparticle penetration, formulation retention, and therapeutic efficacy under pathophysiological conditions.

In summary, this review constructs an integrated framework for efficient therapy of oral mucosal diseases, spanning from pathological mechanisms to clinical translation. The framework begins with pathological features, identifying high recurrence and malignant progression as fundamental drivers for local drug delivery. It then dissects three major delivery barriers, namely salivary flow interference, epithelial tight junctions, and dynamic biomechanical forces. Based on these, formulation strategies are proposed, encompassing wet adhesive material design, penetration enhancement, and mechanical property matching. These strategies are then converted into quantifiable critical quality attributes, such as adhesion strength, release kinetics, penetration depth, and mechanical stability. For the translational phase, we emphasize appropriate preclinical evaluation platforms, ranging from 3D organoid models to microfluidic chips, to overcome interspecies discrepancies. Safety endpoints must be prioritized through systematic toxicological assessment covering cellular, tissue, and organismal levels. Manufacturing challenges, including scale-up, batch consistency, and ambiguous regulatory classification of bioactive materials, must be addressed. Ultimately, all efforts converge on clarifying regulatory and clinical benchmarks, highlighting the need for clearer regulatory guidance and fit-for-purpose evaluation frameworks through multi-stakeholder collaboration. This framework bridges fundamental research and applied translation, offering an actionable roadmap for next-generation oromucosal formulations.

## Figures and Tables

**Figure 1:**
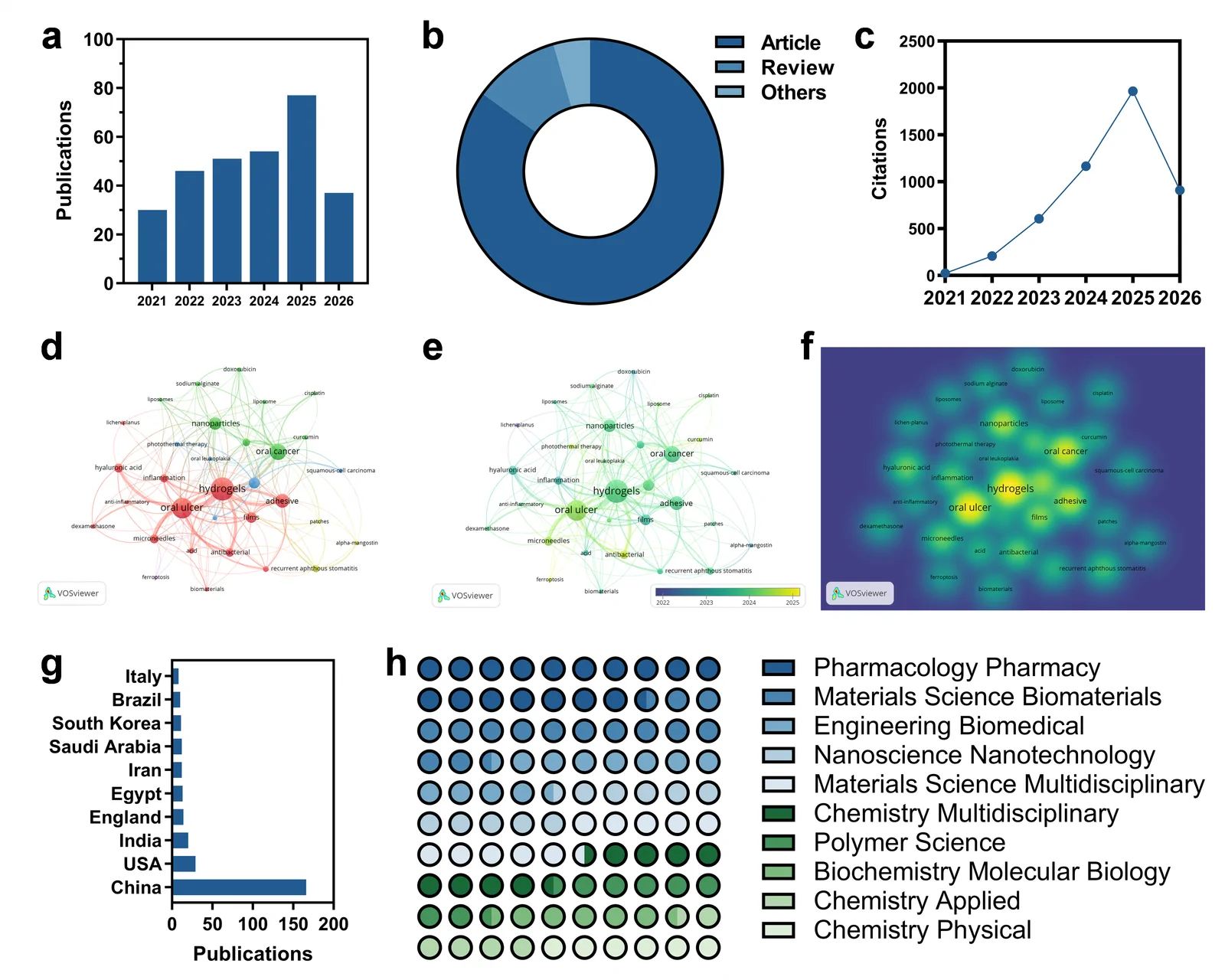
Bibliometric analysis. (**a**) Number of publications versus year. (**b**) Types of publications. (**c**) Citations of these publications. (**d**–**f**) Keyword analysis of 265 research papers using VOSviewer. (**g**) Number of publications from top-10 regions. (**h**) Number of publications in top-10 periodicals.

**Figure 2:**
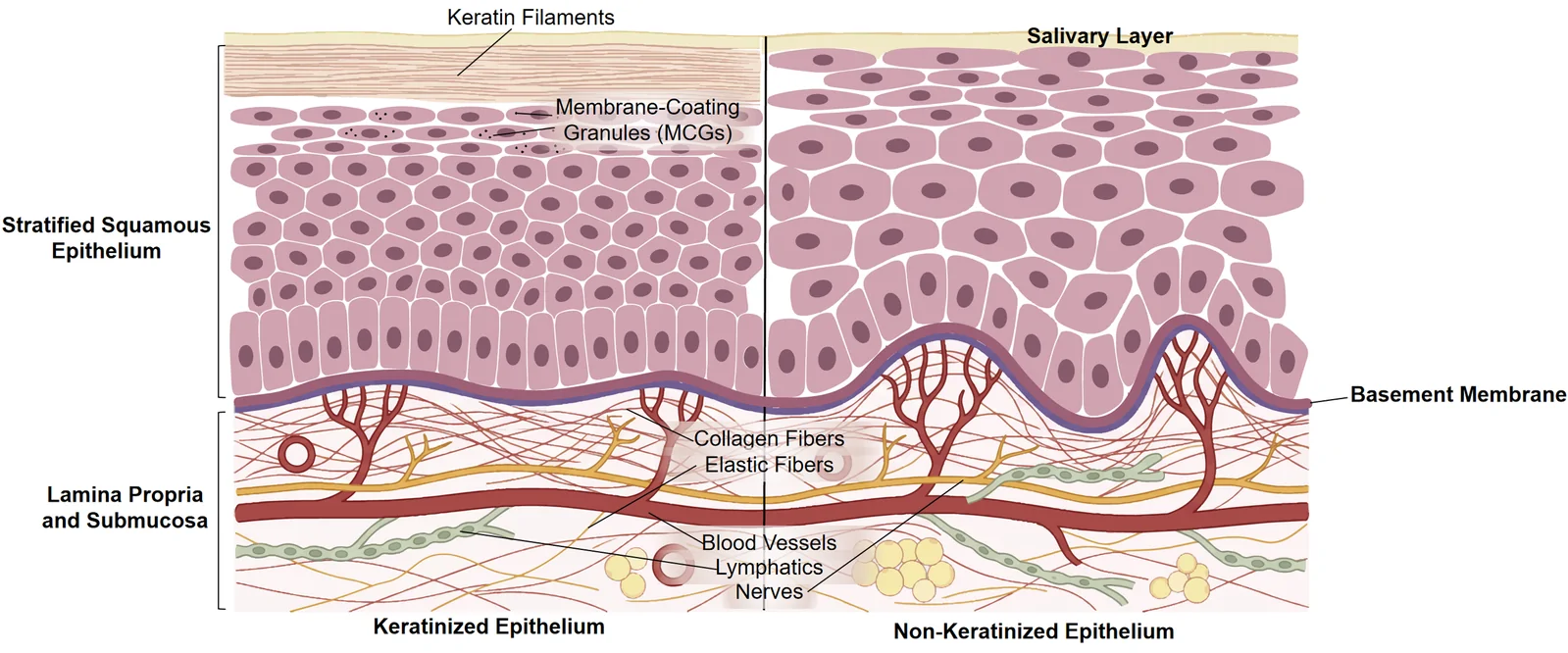
Anatomical structure of the oral mucosa (created by Adobe Illustrator 2024, a vector graphics design software developed by Adobe Inc. (registered in the State of California, USA).).

**Figure 9:**
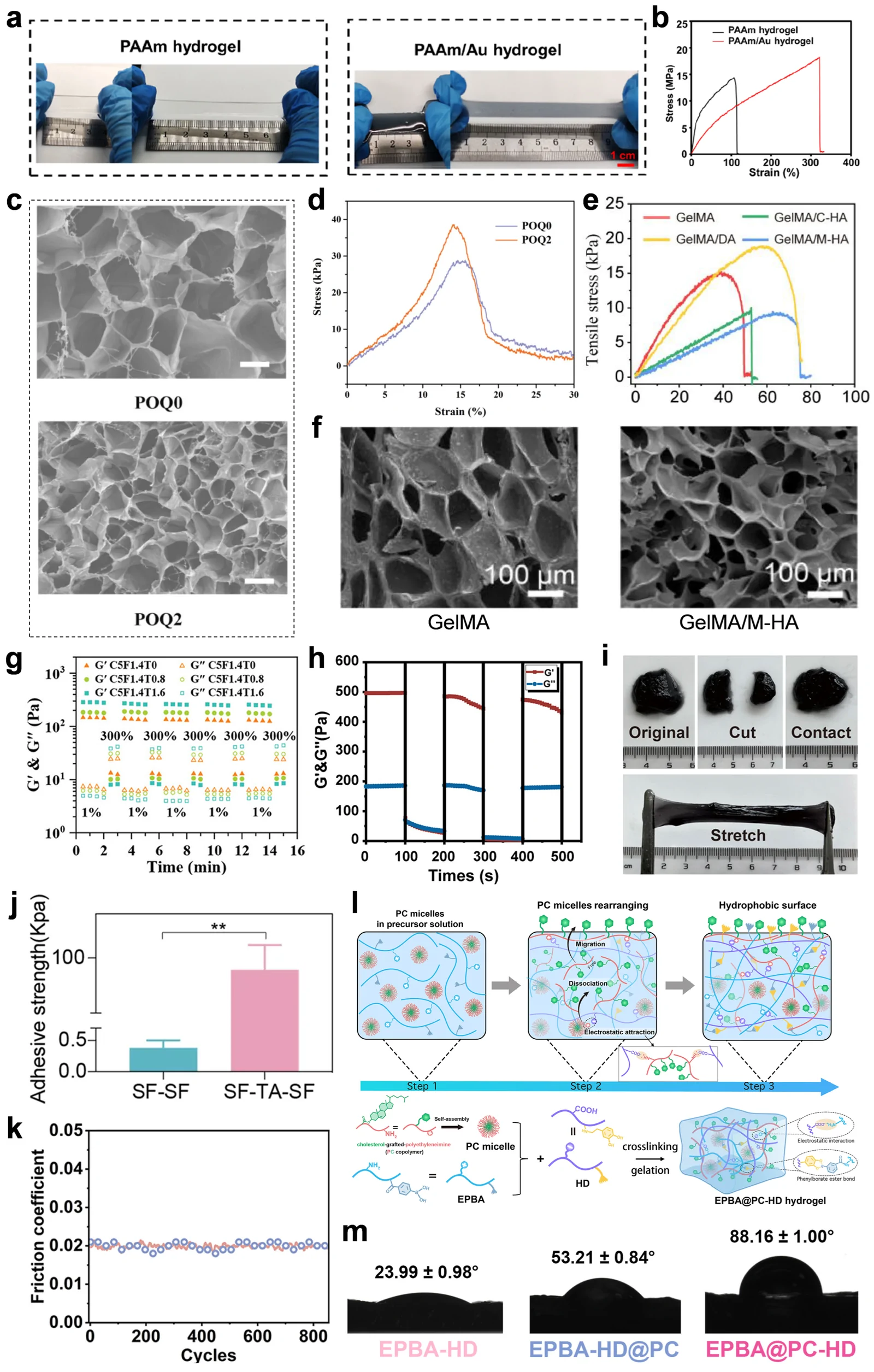
Strategies for enhancing mechanical properties, self-healing properties, and avoiding adhesion to non-target tissues. (**a**) Photographs showing PAAm hydrogel and PAAm/Au hydrogel under tensile loading. (**b**) Tensile stress versus strain curves for PAAm hydrogel and PAAm/Au hydrogel. (**c**) SEM images of POQ0 and POQ2 with a scale bar of 25 μm. (**d**) Lap shear test results of POQ0 and POQ2 hydrogels. (**e**) Compressive–stress curves obtained from different hydrogels. (**f**) SEM images of GelMA and GelMA/M-HA hydrogels. Panels (**a**,**b**), panels (**c**,**d**) and panel (**e**,**f**) adapted with permission from Refs. [112,113,115]. Copyright © Adapted from Elsevier. (**g**) Complex viscosity sweeps of the CFT hydrogels. Panel (**g**) adapted with permission from Ref. [116]. Copyright © Adapted from John Wiley and Sons. (**h**) Continuous alternating strain scan of Fe-TA@P(AM-AA) hydrogels. (**i**) Images depicting the self-healing behavior of Fe-TA@P(AM-AA) hydrogels. Panels (**h**,**i**) adapted with permission from Ref. [117]. Copyright © Adapted from American Chemical Society. (**j**) Interfacial adhesion between an SF-TA film and an SF film. Error bars represent standard deviation (** *p* < 0.01). Panel (**j**) adapted with permission from Ref. [120]. Copyright © Adapted from Frontiers. (**k**) Time-dependent variation in coefficient of friction (COF) for the lubricating layer under water lubrication. (Reciprocating motion, Sliding speed: 30 mm/min). (**l**) Schematic illustration of the fabrication strategy of wet-adhesive EPBA@PC-HD hydrogel. This involves incorporating biocompatible cholesterol micelles into a catechol-modified polyelectrolyte network. The mechanism includes electrostatic attraction-induced dissociation of cholesterol-containing micelles and the consequent reorganization of hydrophobic Chol groups on the hydrogel surface. (**m**) Water contact angle images of EPBA-HD, EPBA-HD@PC, and EPBA@PC-HD hydrogels. Panel (**k**) and panels (**l**,**m**) adapted with permission from Refs. [118,119]. Copyright © Adapted from Elsevier.

**Table 1:** Clinical comparison of recurrent aphthous ulcers, diabetic oral ulcers, treatment-related oral mucositis and oral leukoplakia.

Diseases	Characteristic	Pathogenesis	Mucosal Barrier	Therapeutic Needs	Refs.
Recurrent aphthous ulcers	Common oral mucosal disease	Multifactorial etiology, involving genetic susceptibility, immune system dysfunction, local oral trauma, microbial dysbiosis, and food sensitivity	Abnormal proliferation of opportunistic pathogens directly compromises epithelial integrity, and dysbiosis of oral microbiota contributes to the pathological process of RAU via multiple pathways	Rapid pain relief, promotion of epithelial healing, and modulation of local immune microenvironment	[22,32]
Diabetic oral ulcers	Oral manifestation of impaired wound healing in diabetes, characterized by delayed healing	Hyperglycemic microenvironment impedes ulcer healing through multiple mechanisms (including impaired immune cell function, microangiopathy, and iron metabolism dysregulation), compromising defense against oral microbes	Inhibits keratinocyte migration and significantly delays the re-epithelialization of oral mucosal wounds; microangiopathy leads to basement membrane thickening, limiting oxygen and nutrient delivery to the wound site	Anti-inflammation, ROS scavenging/antioxidation, promotion of angiogenesis, and antimicrobial activity	[24,25,33,34]
Treatment-related oral mucositis	A prevalent disease issue in cancer treatment, characterized by high incidence and delayed healing	Antineoplastic agents/radiotherapy directly inhibit DNA replication and epithelial proliferation, disrupting basal cell renewal, leading to atrophy and ulceration; five-stage biological model applies	Basal epithelial cells are directly damaged by radiotherapy/chemotherapy; reduced epithelial renewal results in mucosal atrophy and ulceration	Concurrent prevention and treatment, wound coverage, deep tissue penetration, basal cell protection, and inflammation reduction	[26,35,36,37]
Oral leukoplakia	Precancerous lesion/oral potentially malignant disease	Product of interaction between environmental carcinogens and genetic susceptibility, involving accumulation of gene mutations induced by chronic tobacco and alcohol exposure	Abnormal epithelial keratinization and differentiation (hyperkeratosis, acanthosis, epithelial dysplasia)	Prevention/reversal of malignant transformation; lesion elimination (surgical excision as gold standard)	[38,39]

**Table 2:** Pharmacological treatment system for oral mucosal diseases and its existing dosage forms.

Drug Therapy System	Specific Route of Administration and Dosage Form	Advantages	Limitations	Clinical Significance	Refs.
Systemic administration	Oral administration (tablets, capsules, suspensions, etc.)	Convenient, safe, economical. High patient compliance	Significant first-pass effect, slow onset	Suitable for chronic diseases requiring long-term stable blood concentration (e.g., autoimmune diseases) or non-urgent oral infections	[13,45,46]
	Injection (IV, IM, SC)	High bioavailability, dose controllable	Invasive procedure, risk of systemic side effects, poor convenience	Primarily used for oral mucosal diseases that are ineffective with local treatment, extensive multi-site, or refractory/severe conditions, such as acute severe episodes of oral lichen planus, as well as deep space infections requiring rapid control of the condition	
Local administration	Liquid dosage forms (mouthwash, spray)	Convenient to use, high safety	Short retention time	Suitable for widespread superficial inflammation or infection, e.g., mouthwash treatment of oral candidiasis, prophylactic mouthwash for radiation-induced oral mucositis	[13,16,43,44,47]
	Semi-solid dosage forms (gel, ointment)	Better adhesion than liquids	Poor comfort, may be diluted by saliva	Commonly used for localized oral ulcers, erosive lichen planus	
	Solid dosage forms (patch, film)	Good targeting and persistence, improved comfort	Difficult to adapt to the dynamic oral environment	Suitable for single localized lesions requiring prolonged, high-concentration drug action, e.g., major aphthous ulcers	

**Table 3:** Commercially available local formulations (approved by the U.S. FDA) for oral mucosa diseases (From 2000 to 2026).

Product Name	FDA Approval Date	Primary Indications	Formulation Type	Official FDA Verification Channels
THE SALICEPT ORAL PATCH	3 October 2001	Recommended for use in extraction sites and management of alveolar osteitis (dry socket), and also intended for the management of all types of wounds, injuries, and ulcers of the oral mucosa	Freeze-dried hydrogel	FDA 510(k) Number: K012126
GELCLAIR CONCENTRATED ORAL GEL	21 December 2001	Indicated for pain management for oral mucositis, aphthous ulcers, and traumatic ulcers induced by radiotherapy and/or chemotherapy	Concentrated oral gel	FDA 510(k) Number: K013056
TRADENAME (amlexanox) Mucoadhesive Patch	29 September 2004	Indicated for recurrent aphthous ulcers in immunocompetent individuals aged 12 years and older	Oral mucosa-adherent patch	FDA Drugs@FDA: NDA 21-727
MUGARD MUCOADHESIVE ORAL WOUND RINSE	11 February 2006	Indicated for oral mucositis, aphthous ulcers, and traumatic ulcers induced by radiotherapy and/or chemotherapy	Mucoadhesive oral wound rinse	FDA 510(k) Number: K062795
ORAVIG (miconazole)	16 April 2010	Indicated for the local treatment of oropharyngeal candidiasis (OPC) in adults	Mucosal adhesive buccal tablet	FDA Drugs@FDA: NDA 22-404
EPISIL	13 September 2011	Indicated for pain relief for various oral lesions such as oral mucositis caused by radiotherapy and/or chemotherapy	Oromucosal liquid	FDA 510(k) Number: K101769
SITAVIG (acyclovir) Buccal Tablet	12 April 2013	Indicated for the treatment of recurrent herpes labialis (cold sores) in immunocompetent adults	Buccal tablet	Drugs@FDA: NDA 203791
Silatrix Oral Gel	20 November 2020	Indicated for the management of oral mucosal lesions (including aphthous ulcers, mucositis, traumatic ulcers, and denture-induced abrasions)	Amorphous hydrogel	FDA 510(k) Number: K202000
Curajel™ Oral Gel	15 June 2026	Indicated for use with all types of ulcers and small wounds of the oral mucosa, including canker sores, aphthous ulcers, and denture-induced abrasions	Amorphous hydrogel	FDA 510(k) Number: K260126

The formulation information in Table 2 can be queried at the following URL: Drugs@FDA https://www.accessdata.fda.gov/scripts/cder/daf/ (accessed on 4 September 2026); FDA 510(k) https://www.accessdata.fda.gov/scripts/cdrh/cfdocs/cfPMN/pmn.cfm (accessed on 4 September 2026).

**Table 4:** Strategies, methods, and measures for next-generation oroucosal formulations adapting to complex oral environments.

	Strategy	Method	Measure	Refs.
New-generation oromucosal formulation	Strategies for enhancing adhesion	Eliminate the interference of saliva	Dry formulation, hydrophobic interactions	[100,101,102]
		Enhance the interaction with the tissue and mucus layer	Hydrogen bonds, electrostatic interactions, Schiff bases, amide bonds, disulfide bonds, etc.	[23,103,104,105,106]
	Strategies for enhancing penetration	Permeation enhancer	Surfactants, bile salts, fatty acids, etc.	[107,108]
		Novel methods for enhancing permeability	Microneedles, nanoparticle delivery system	[109,110,111]
	Strategies for enhancing adaptability to mechanical environments	Improve mechanical properties	High-modulus materials, crosslinking density	[112,113,114,115]
		Enhance self-healing properties	Dynamic chemical bonds, reversible physical interactions	[116,117]
		Avoid adhesion to non-target tissues	Asymmetric surface	[118,119,120]

**Table 5:** Mechanism of action, common concentrations, permeability enhancement efficacy, and toxicity of traditional permeability-enhancing agents.

Enhancer Classification	Working Mechanism	Typical Concentration Range	Level of Enhancement	Barrier Disruption	Mucosal Toxicity	Refs.
Surfactant	Isolating lipids from intercellular spaces and cell membranes; generating self-assembled architectures; disassembling tight junctions.	Above the CMC, a marked permeation enhancement is observed.	Strong	Strong	Damages the mucosal cell layer	[166,171]
Bile salt	Formation of micelles and reverse micelles; disruption of tight junctions; reduction in mucus viscoelasticity	Typically ranges from 2 mM to 10 mM	Strong	Strong	Swelling; epithelial layer injury; separation of epithelium from connective tissue	[162,172,173,174]
Fatty acids	Interferes with the arrangement of lipids in oral epithelial cells; enhances lipid distribution	0.5–15%	Medium	Weak	Burning sensation, sour taste, or bitter taste at high concentrations	[165]
CPPs	Direct penetration; triggering endocytosis	1 nM	Strong	Weak	No significant toxicity	[175,176,177]

## Data Availability

No new data were created or analyzed in this study. Data sharing is not applicable to this article.

## Abbreviations

The following abbreviations are used in this manuscript:

BLA	Biologics License Application
CIOM	Chemotherapy-Induced Oral Mucositis
COX-2	Cyclooxygenase-2
CPC	Cetylpyridinium Chloride
CPPs	Cell-Penetrating Peptides
CS	Chitosan
DA	Dopamine
DMPA	2,2-dihydroxymethylpropionic Acid
DOPA	3,4-dihydroxy-L-phenylalanine
DOPG	Dioctylphosphatidylglycerol
DOX	Doxorubicin
EPR	Enhanced Permeability and Retention
FDA	Food and Drug Administration
GCS	Glycol Chitosan
GHS	Globally Harmonized System
HA	Hyaluronic Acid
IPN	Interpenetrating Polymer Network
LZM	Lysozyme
MCGs	Membrane-Coating Granules
ML	Machine Learning
MN	Microneedles
NDA	New Drug Application
NHS	N-Hydroxysuccinimide
OC	Oral Candidiasis
OLK	Oral Leukoplakia
OLP	Oral Lichen Planus
OPC	Oropharyngeal Candidiasis
OSCC	Oral Squamous Cell Carcinoma
PAAm	Polyacrylamide
PEI	Polyethylenimine
PGE2	Prostaglandin E2
PMOA	Primary Mode of Action
PS-80	Polysorbate 80
PVA	Polyvinyl Alcohol
RAU	Recurrent Aphthous Ulcers
RIOM	Radiation-Induced Oral Mucositis
SEDDS	Self-Emulsifying Drug Delivery System
SEM	Scanning Electron Microscopy
SF	Silk Fibroin
SLS	Sodium Lauryl Sulfate
SRGP	Sprayable Rapid Gelation Powder
STC	Sodium Taurocholate
UV	Ultraviolet
